# Development of an in situ simulation-based continuing professional development curriculum in pediatric emergency medicine

**DOI:** 10.1186/s41077-020-00129-x

**Published:** 2020-07-01

**Authors:** James S. Leung, Mandeep Brar, Mohamed Eltorki, Kevin Middleton, Leanne Patel, Meagan Doyle, Quang Ngo

**Affiliations:** 1 Division of Pediatric Emergency Medicine, Department of Pediatrics, McMaster University, Health Sciences Centre, Room 2R014, 1280 Main Street W, Hamilton, ON L8N 3Z5 Canada; 2 Emergency Department – McMaster Children’s Hospital, Hamilton Health Sciences, Hamilton, ON Canada; 3 Simulation and Outreach, McMaster Children’s Hospital, Hamilton, Canada; 4 Department of Pediatrics, McMaster University, Hamilton, Canada

## Abstract

**Background:**

Continuing professional development (CPD) activities delivered by simulation to independently practicing physicians are becoming increasingly popular. At present, the educational potential of such simulations is limited by the inability to create effective curricula for the CPD audience. In contrast to medical trainees, CPD activities lack pre-defined learning expectations and, instead, emphasize self-directed learning, which may not encompass true learning needs. We hypothesize that we could generate an interprofessional CPD simulation curriculum for practicing pediatric emergency medicine (PEM) physicians in a single-center tertiary care hospital using a deliberative approach combined with Kern’s six-step method of curriculum development.

**Methods:**

From a comprehensive core list of 94 possible PEM clinical presentations and procedures, we generated an 18-scenario CPD simulation curriculum. We conducted a comprehensive perceived and unperceived needs assessment on topics to include, incorporating opinions of faculty PEM physicians, hospital leadership, interprofessional colleagues, and expert opinion on patient benefit, simulation feasibility, and value of simulating the case for learning. To systematically rank items while balancing the needs of all stakeholders, we used a prioritization matrix to generate objective “priority scores.” These scores were used by CPD planners to deliberately determine the simulation curriculum contents.

**Results:**

We describe a novel three-step CPD simulation curriculum design method involving (1) systematic and deliberate needs assessment, (2) systematic prioritization, and (3) curriculum synthesis. Of practicing PEM physicians, 17/20 responded to the perceived learning needs survey, while 6/6 leaders responded to the unperceived needs assessment. These ranked data were input to a five-variable prioritization matrix generating priority scores. Based on local needs, the highest 18 scoring clinical presentations and procedures were selected for final inclusion in a PEM CPD simulation curriculum. An interim survey of PEM physician (21/24 respondents) opinions was collected, with 90% finding educational value with the curriculum. The curriculum includes items not identified by self-directed learning that PEM physicians thought should be included.

**Conclusions:**

We highlight a novel methodology for PEM physicians that can be adapted by other specialities when designing their own CPD simulation curriculum. This methodology objectively considers and prioritizes the needs of practicing physicians and stakeholders involved in CPD.

## Background

Independently practicing physicians have a moral imperative to commit to continuing professional development (CPD) in order to provide high-quality patient care and maintain ongoing public trust as a self-regulated profession [1]. The current conceptualization of CPD emphasizes updating and acquiring all of the broad competencies required for practicing high-quality medicine, including continuing medical education (CME), whereby technical knowledge and skills are refined and acquired [2], as well as the physician’s capacity as a communicator, collaborator, leader, health advocate, and professional [3]. In recent years, the increasing emphasis on improving healthcare quality and patient safety has transformed the needs and expectations of medical education provided to physicians in training and in practice [4, 5].

There is mounting public pressure for maintenance of certification (MOC) programs to support activities demonstrating change in physician behavior and improving patient outcomes rather than focusing on individual learning outcomes [6]. Concurrently, there has been mounting pressure from physicians for MOC programs to demonstrate value, encouraging CPD delivery in novel, impactful methods with high-quality learning outcomes. In Canada, specialists are mandated to participate in the Royal College of Physicians and Surgeons of Canada (RCPSC) MOC program. A 2016 cross-sectional survey of the RCPSC MOC program found a perceived lack of impact of the program on physician learning and perception from physicians that the MOC program served as a monitoring/regulatory body rather than its intended purpose as a mechanism to enhance lifelong learning and reflection [7]. A similar, US survey in 2016 revealed physicians desire more practice-relevant learning that is time-efficient, low-cost, and with topics of their choosing [8].

### Rationale for simulation in continuing professional development

Evidence from several studies [7–12] suggests three factors consistently influence CPD learning success: accurate needs assessments prior to the learning activity, interaction amongst physician learners with opportunities to practice, and multifaceted educational activities [13]. Simulation-based medical education (SBME) addresses all three of these factors and has a greater influence on physician learning outcomes and practice compared to the traditional CPD methods such as lectures and conferences [12, 13]. SBME offers a number of potential advantages over traditional educational methods including simultaneously addressing CME needs and advanced knowledge domains such as communication and teamwork, direct observation of clinical performance with feedback through debriefing, and the capability to practice medical procedures and methods with minimal patient risk. SBME is also unique in its ability to train physicians to operate within interprofessional healthcare teams and the larger systems in which they function.

For these reasons, MOC programs are increasingly embracing SBME for CPD. In Canada, a 2009 RCPSC MOC program evaluation led to simulation being recognized as a method of practice assessment. In the US, SBME is required for primary licensure of anesthesiologists and surgeons and is utilized for CPD with anesthesia, surgery, internal medicine, family medicine, emergency medicine, pediatrics, and radiology [14]. There is strong physician demand for SBME, with 46% of practicing US physicians wanting more simulation activities for CPD in a 2016 national cross-sectional survey [8].

There is less understanding regarding the effectiveness of SBME for CPD, as most published literature focuses upon trainee physicians. However, evidence is emerging to support SBME effectiveness for board-certified physicians. A systematic review of 39 available studies in 2015 revealed benefits of SBME for CPD in acute care physician’s self-reported skills and attitudes, and immediate and sustained improvements in physician educational outcomes [5]. As an educational method rooted in constructivism, SBME theoretically has a greater potential educational benefit for practicing physicians due to the presence of pre-existing clinical experiences to build upon. With this potential educational impact, understanding methods to optimize SBME for CPD is imperative.

### Rationale for developing a curriculum for simulation CPD

In contrast to medical trainee programs, CPD programs are distinguished by lack of specific, pre-defined curricula and emphasis on self-identified learning interests. However, evidence has shown that despite being a key motivator for ongoing engagement [8, 15], self-direct learning (SDL) is insufficient for a robust CPD program as physicians are unable to accurately self-assess learning needs meeting professional and societal demands [16]. A curriculum including necessary topics not identified by SDL would create a more robust and effective CPD program.

At present, much of our experience with SBME and CPD consists of ad hoc case creation suiting SDL needs of physicians or as a reaction to systems needs (i.e., critical safety events). This approach limits the educational potential of SBME for CPD. In contrast, a simulation curriculum, planned in advance from perceived and unperceived learning needs, is more robust, sustainable, and proactive for healthcare system needs. We hypothesize that such a curriculum would be non-inferior in educational effectiveness compared to the traditional approach.

Curriculum integration prospectively distributes CPD learning over several sessions, broadening the scope of SBME and allowing deliberate practice and spaced repetition of targeted objectives to achieve mastery learning [17]. In particular, we feel spaced repetition is powerful for equally important learning objectives such as communication, which can be repeating educational aims, in a curriculum with various clinical presentations. From the perspective of a MOC program, a curriculum vastly improves simulation logistics and operations. Although seemly trivial, prior CPD literature has identified logistical factors as a leading facilitator and barrier to successful and sustainable CPD [10]. A curriculum serves to assist course facilitators with more predictable planning, known costs, lead-time for quality simulation scenario development and testing, and standardization of local practice. These changes potentially translate to an overall higher quality educational experience.

Conceptually, we envision a curriculum whereby a group of board-certified physicians shares in completing a comprehensive series of longitudinally delivered simulation scenarios. Using appropriate knowledge/experience dissemination tools, this approach is rooted in the educational theories of Experiential Learning, Social Learning and Communities of Practice [18–21]. This co-produced curriculum approach is a pragmatic solution to allow a greater variety of cases to be covered in a feasible timeframe than a single individual completing the curriculum alone.

### Our aim: development of simulation CPD curriculum for PEMs

In pediatric emergency medicine (PEM), simulation is vital for practicing physicians as the frequency of critically ill children is low relative to the total volume of children presenting to pediatric emergency departments [22]. Simulation allows PEM physicians to refine and retain resuscitative skills to remain prepared to manage rare, high stakes events. The tremendous breadth of clinical presentations in PEM creates both a challenge and rationale for a well-designed CPD simulation curriculum. An objective and systematic methodology using input from vital stakeholders to select the important clinical presentations and procedures to include in such a comprehensive curriculum is critical.

To design our simulation CPD curriculum, we formed an Interprofessional PEM Simulation Oversite Committee (PEMSOC) with membership consisting of local physicians, a registered nurse (RN), pharmacist, and a simulation operation specialist with a professional background as a registered respiratory therapist (RRT). The overarching aim of our simulation CPD program was to utilize simulation to develop and maintain the clinical skills of health care providers with the goal of providing world-class quality care to children. The primary objective of this paper is to describe our methods for the development of a SBME curriculum for CPD of practicing PEM physicians.

## Methods

### Literature review

To develop our curriculum, we first looked at existing literature on CPD simulation curriculum design. We conducted a literature review on curriculum design methodology for simulation and CPD. A formal search by a research librarian conducted on February 14, 2019, revealed 172 results when including articles (Epub Ahead of Print, In-Process & Other Non-Indexed Citations) from OVID Medline between 1946 and February 14, 2019, with the following MESH terms: Professional Competence (MESH 108801) AND Simulation Training/or exp Computer Simulation/ or exp Patient Simulation (MESH 222646) AND faculty, medical/ed or “Education, Medical, Continuing” (MESH 24165). A manual review of these 172 results revealed 0 articles referencing a curriculum design methodology for physician CPD using simulation.

### Curriculum design process

With no identified pre-existing literature, we endeavored to develop our own methodology for CPD simulation curriculum design. Although our methodology could be considered for general CPD curriculum design, our focus was to develop a curriculum optimized for simulation as the chief educational strategy.

To develop our methodology, we adapted Kern’s [23] established approach to curriculum design for a novel application of simulation curriculum design with a CPD audience. We completed the following six steps: (1) problem identification by a general needs assessment, (2) targeted needs assessment, (3) goals and specific measurable objectives, (4) educational strategies (SBME in our case), (5) implementation, and (6) evaluation and feedback [24] This process was followed by a deliberative curriculum approach with experts determining the final curriculum content. Specific deliberative considerations included lack of standardized core learning objectives, greater variations in learner clinician experience and practice patterns, non-static learner composition, need for voluntary learner participation, medico-legal ramifications of simulations, and psychological safety considerations of participants such as reputation.

**Fig. 1:**
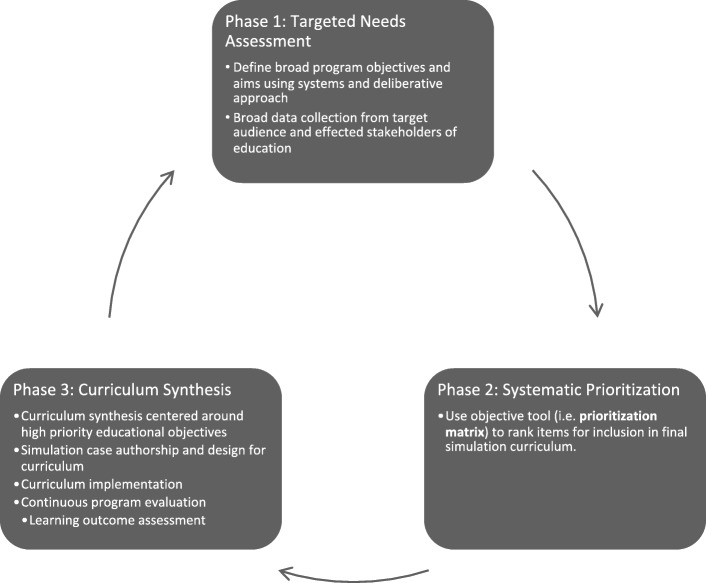
Continuing professional development simulation curriculum design process. A three-phase curriculum development process adapted from Kern’s curriculum development approach used to generate a simulation curriculum for continuing professional development (CPD) for pediatric emergency medicine physicians. Phase 1 begins with a detailed targeted needs assessment involving all relevant stakeholders of the physician’s continuing professional development. Phase 2 follows with systematic prioritization of learning topics to include in the curriculum using data collected from the targeted needs assessment. A prioritization matrix is used to rank items for curriculum inclusion. Finally, in phase 3, selected learning topics are organized by educational experts into a curriculum to be implemented and evaluated. These three phases can be repeated in a cyclical manner as the curriculum is refined and reimagined over time

We consolidated the above processes into our novel systematic three-phase methodology described below (Fig. 1: Continuing professional development simulation curriculum design process).

#### Phase 1: systematic and deliberative needs assessment

We conducted a detailed general and targeted needs assessment using a systems and deliberative approach, including perceived and unperceived learning needs from our target learner audience.

A perceived needs assessment was completed using a 20-item electronically distributed survey (SurveyMonkey Inc., San Mateo, CA, USA, www.surveymonkey.com) sent to all 20 practicing PEM physicians at our institution from March 28, 2018, to April 11, 2018. The primary objective of this survey was to determine topics our audience wished to cover in simulations. The secondary objective focused on local simulation etiquette and tailoring learning processes for our target audience. Participants were presented a comprehensive list of 65 clinical presentations and 29 critical procedures from the 2013 RCPSC Objectives of Training in pediatric emergency medicine [25] and asked if they felt an educational need to address by an educational simulation session. This document, originating from our national board-certifying organization, was selected as the foundational list as we felt it best represented the competencies expected of PEM physicians upon entry into practice. The survey was anonymously completed by 17 physicians (85% response rate), with results found in Additional file 1: Needs assessment survey.

Considering the aforementioned limitations of SDL in identifying learning needs, we conducted an unperceived needs assessment by asking our hospital leadership to rank on a 5-point Likert scale (1 = low priority, 3 = moderate priority [default starting score], 5 = high priority) from the same expansive list of clinical presentations and procedures presented to PEM physicians from the perspective of their position of leadership. Six positions of leadership polled were multi-disciplinary and composed of our PEM medical director and division head, deputy division head, PEM division safety lead, general emergency department site lead at our local tertiary care general hospital, PEM pharmacist lead, and PEM RN lead and trauma coordinator. All six leaders (100% response rate) completed the survey. In addition to leadership needs, PEMSOC accounted for patient-level needs by reviewing cases discussed from the previous 2 years of PEM divisional quality improvement and patient safety rounds (QI/PS). PEMSOC reviewed 21 clinical presentations but did not go into an in-depth chart review of each case at this stage, as our focus was to determine topics to cover in the simulation curriculum.

#### Phase 2: systematic prioritization

In order to objectively synthesize all of our collected needs assessment data, we utilized a prioritization matrix to objectively determine the clinical presentations and critical procedures to include in our PEM simulation CPD curriculum. While less commonly utilized in medical education and simulation, prioritization matrices have been applied in healthcare quality improvement [26, 27] and are commonly utilized in business and military goal setting where resources are limited and multiple objectives need to be addressed [28]. As there are several published matrices underpinned by different mathematical algorithms, we adapted an available prioritization matrix utilized by members in other local educational projects.

Our prioritization matrix utilized five data categories determined by PEMSOC a priori: perceived needs assessment results, unperceived needs assessment results, feasibility of conducting the simulation, benefit to patients if practiced, and educational value of simulating the case for learning (balanced with frequency of encountering the case in real clinical practice, in which case there would be less educational need). Ranked data ranging from scores of 1 (low rank) to 5 (high rank) were input into the matrix for each clinical presentation and procedure and each data category, generating an overall priority score which was utilized by PEMSOC in consideration for curriculum inclusion (Table 1).

**Table 1:**
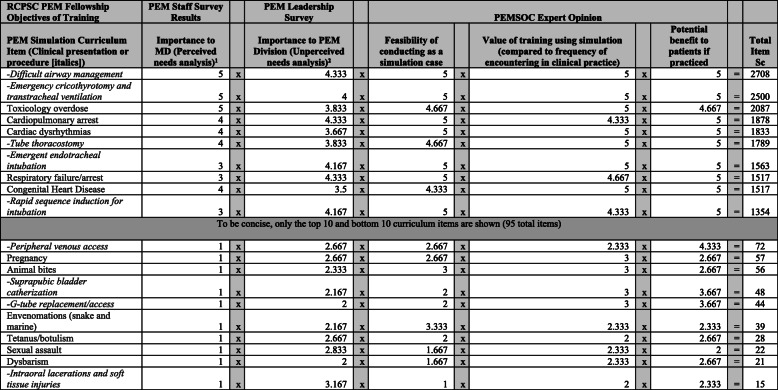
Pediatric emergency medicine continuing professional development simulation curriculum systematic prioritization matrix

All items are ranked from 1 (low priority) to 5 (high priority), with 3 (moderate priority) being starting default score

*RCPSC* Royal College of Physicians and Surgeons of Canada, *PEM* pediatric emergency medicine, *PEMSOC* Pediatric Emergency Medicine Simulation Oversite Committee

^1^Proportion of PEM physicians within the division feeling that curriculum item should be included in CPD simulation curriculum: 1 = 0–20% PEM physicians responding yes; 2 = 21–40%; 3 = 41–60%; 4 = 61–80%; and 5 = 81–100%

^2^Mean rank from 1–5 from the following 6 PEM leaders: medical director and division head, deputy division head, PEM quality improvement and patient safety lead, general emergency medicine site lead, PEM pharmacy lead, PEM nursing lead

To complete our perceived needs assessment data category, we recorded the frequency of “yes” responses from all 17 survey participants, converting individual qualitative responses (nominal/binary data) into quantitative discrete frequency data. These frequency data were categorically assigned a ranking score from 1 to 5 (1 = 0–20%; 2 = 21–40%; 3 = 41–60%; 4 = 61–80%; 5 = 81–100%) and inserted into the prioritization matrix. With the unperceived needs assessment category, we input the mean ranking score (from 1 to 5) of all six leaders into the priority matrix. For the remaining three data categories, PEMSOC members independently assigned a ranking score from 1 to 5 (1 = low priority, 3 = moderate priority [default starting score], 5 = high priority) for each data category, with the mean score input into each respective column in the prioritization matrix. PEMSOC committee members also included the 21 QI/PS rounds cases by factoring their scores for the “benefits to patient” data category of the matrix. As PEMSOC is comprised of inter-professional education and simulation specialists with formal training (simulation, quality improvement, Master of Education, curriculum design), these data represent the expert opinion necessary for pragmatic grounding we felt necessary for eventually creating a feasible, high-quality curriculum.

#### Phase 3: curriculum synthesis, implementation, and continuous evaluation

With these data, the matrix generated priority scores for PEM clinical presentations and critical procedures. These scores were organized into two ranked lists: a clinical presentation list and a critical procedure-list based on priority score (Table 2). Each list was manually reviewed by PEMSOC to ensure overlapping or duplicate items were combined into a single item.

**Table 2:**
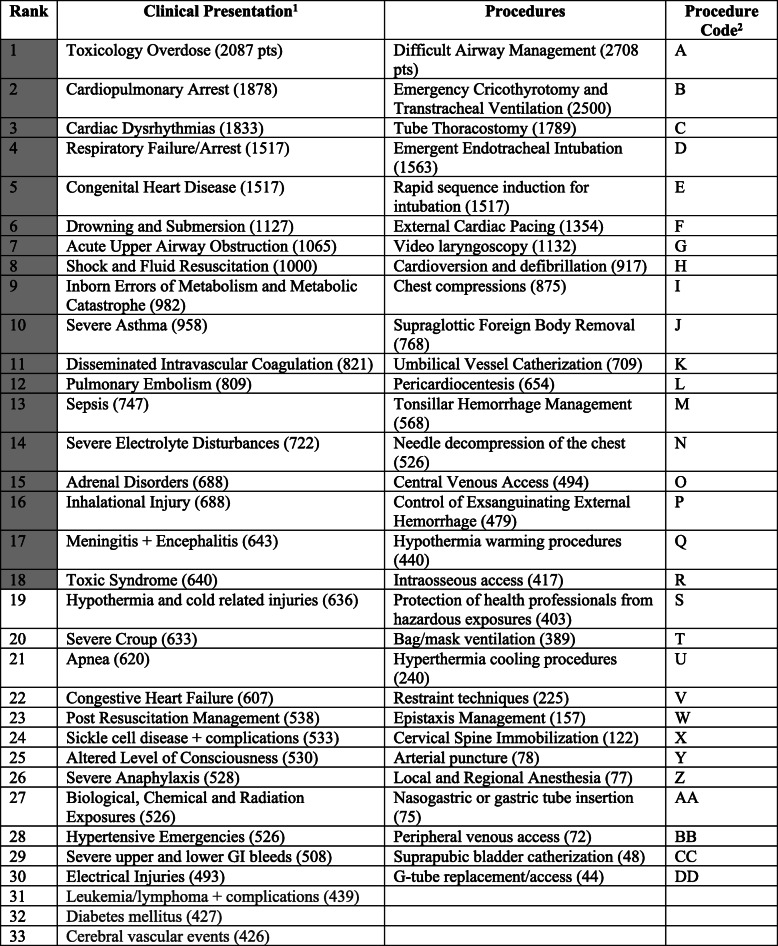
Ranked scores for pediatric emergency medicine continuing professional development simulation curriculum generated by priority matrix

Clinical presentations ranked 1–18 and procedures codes A–R were selected for inclusion in the final curriculum

^1^Clinical presentations related to trauma were omitted from our needs assessment and hence systematic prioritization as they were considered beyond the scope of our continuing professional development program and encompassed in a different trauma simulation program at our institution

^2^Please use this column when referencing procedures found in Table 3. Pediatric emergency medicine continuing professional development simulation curriculum

Based on expert opinion, factoring the large number of items to cover, balanced with the need for regular curriculum refresh and review, PEMSOC decided on a 24-month curriculum block, filled with an 18-simulation scenario curriculum, with one scenario per month. We deliberately planned 6 months of “flex-time” to maintaining flexibility to repeat high-yield scenarios, conduct urgent simulation cases (e.g., to address urgent patient safety and quality improvement needs), and reschedule the unanticipated events where a simulation sessions are cancelled.

With the number of cases to include for our single-center’s needs decided, PEMSOC selected the highest-scoring 18 clinical presentations and 18 critical procedures objectively determined by the matrix for inclusion in the CPD curriculum. PEMSOC mapped individual clinical presentations to critical procedures to synthesize our 18-simulation scenario curriculum (Table 3). We centered our scenarios around clinical presentations, as our intention was to cover all procedures at least once in the curriculum with repetition of high-yield procedures for mastery learning. Learning objectives for each case were set by PEMSOC based on expert opinion, including learning points from the 21 QI/PS round cases when appropriate. For consistent formatting, all cases were drafted using an internally designed standard simulation template (Additional file 2: Sample simulation scenario).

**Table 3:** Pediatric emergency medicine continuing professional development simulation curriculum

Clinical presentation #	Clinical presentation	Sample case stem	Cross-covered clinical presentations (clinical presentation #)	Key procedures covered in case	Secondary procedures (procedure key code^1^)
1	Toxicology	TCA toxicity*	3, 4, 8, 18	Emergency intubation, rapid sequence induction, cardioversion/defibrillation	R
2	Cardiopulmonary arrest	Isolated blunt chest trauma—cardiac tamponade	6	Pericardiocentesis	D, H, I, R
3	Cardiac arrhythmia	Toxin-induced bradycardia (calcium channel blocker)	1, 4, 14	External cardiac pacing	D, E, H, I, O, R
4	Respiratory failure	Severe pneumonia/empyema	13	Tube thoracostomy	D, E
5	Congenital heart disease	Tetrology of Fallot spell	3	Central venous access	D, E
6	Drowning/submersion	Hypothermic arrest	2, 3, 4	Chest compressions, hypothermia warming procedures	D, H
7	Upper airway obstructions	Complete foreign body airway obstruction	4	Supraglottic foreign body removal	A, B, D, E, G, I
8	Shock and fluid resuscitation	Massive tonsillectomy + adenoidectomy bleed		Tonsillar hemorrhage management, control of exsanguinating external hemorrhage	A, B, D, E, G, Q, R
9	Inborn error of metabolism	Newborn with urea cycle defect	4	Umbilical vessel catherization	D, E
10	Severe asthma	Asthma + respiratory failure + complications	4	Tube thoracostomy, needle decompression	A, B, D, E, G, I
11	Disseminated intravascular coagulation (DIC)	Snake bite/laceration	1, 2, 3	Control of exsanguinating external hemorrhage	
12	Pulmonary embolism (PE)	Massive PE with cardiac effects	2, 3		
13	Sepsis (severe)	Fever in returning traveller + sepsis	4	Central venous access	D, E
14	Severe electrolyte abnormalities	Chronic renal failure requiring dialysis			
15	Adrenal disorders	Adrenal crisis + hyperkalemia	14	Cardioversion and debirillation, chest compressions	
16	Inhalational injury	Caustic ingestion	4	Difficult airway management, emergency cricothyrotomy, emergency cricothyrotomy and transtracheal ventilation, emergent endotracheal intubation, RSI for intubation	O, R
17	Meningitis/encephalitis	Meningitis with increased intracranial pressure and DIC	4, 11	Central venous access	D, E
18	Toxic syndrome	Local anesthetic toxicity	1	Cardioversion and debirillation, chest compressions	D, E

^1^Please reference Table 2, “Procedure key code” column for designated procedures

Our curriculum was incorporated into a monthly pre-existing interprofessional in situ simulation program within our pediatric emergency department starting in April 2019. In our single-center PEM division, all practicing PEM physicians participated in the simulation program, with leadership expecting physicians to participate in one to two simulation sessions within a 24-month period. Prior to our curriculum, cases were selected at random from a database, or created on an ad hoc basis, by request of the participating PEM physician, 1 week in advance of the scheduled simulation session. After curriculum implementation, practicing PEM physicians were instead given an option of three simulation scenarios selected from our 18 case curriculum, 1 week in advance of the simulation session. The selected scenario would be marked as “completed” by PEMSOC and removed from circulation until all cases were covered in the 24-month curriculum block. We allowed PEM physicians to know the clinical presentation in advance to provide context, reduce “performance anxiety” [29], encourage psychological safety [30], and encourage learning in advance of the simulation. We also shared a limited version of the simulation curriculum in Table 3 with all PEM physicians (only detailing “clinical presentation,” “sample case stem,” and “unique procedures covered in case”). This sharing was intended to facilitate learning through community of practice and further promote psychological safety. Other interprofessional participants were also provided the case topic in advance, if asked.

In the simulation session, the PEM physician completed a 1-h in situ interprofessional simulation involving practicing RNs, pharmacists, and RRTs in our pediatric emergency department resuscitation room. Following each simulation, immediate post-event debriefing of the entire interprofessional team was completed by a simulation facilitator with formal debriefing training (PEMSOC member). Key learning points relating to team-level knowledge and performance gaps were addressed during debriefing. Within a month of the simulation session, an information package highlighting key knowledge and practice tips (called “SimBITS” by our team) was created and electronically disseminated by PEMSOC to all PEM, RN, and RRT practitioners in our division (Additional file 3: Sample SimBITS). To protect the psychological safety of simulation participants, the newsletter omitted all identifying information and performance gaps of participants.

As our learner group is expected to be non-static, with evolving clinical experiences and evolving learning needs, we planned to continuously evaluate our simulation program, with repeated curriculum redesign following the above process every 20 months.

## Results

### Interim curriculum feedback

Between November 10 and 15, 2019, PEMSOC distributed an electronic survey to all 24 practicing PEM physicians (SurveyMonkey Inc., San Mateo, CA, USA, www.surveymonkey.com) (our division had grown by four members in the interval time). The primary objective of this survey was to elicit feedback on the curriculum contents, with a secondary objective of determining learner attitudes to our curriculum approach (Additional file 4: Interim feedback survey).

**Fig. 2:**
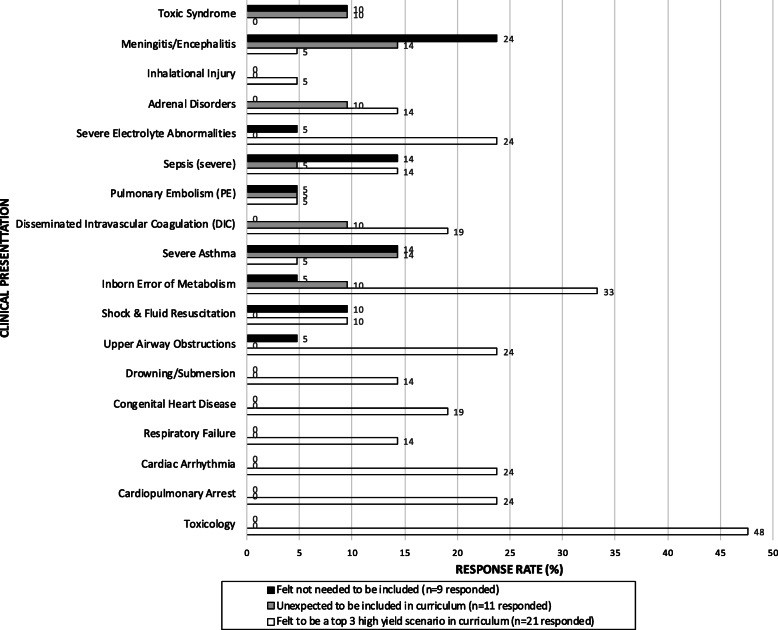
PEMSOC interim curriculum survey results regarding curriculum contents. Pediatric emergency medicine physicians (*n* = 21/24) completed an online survey indicating personal opinions on whether clinical presentations should be included in a simulation curriculum designed for continuing professional development. Clinical presentations are listed in ascending order of priority score in curriculum design process. Clinical presentations with high priority scores (toxicology, cardiopulmonary arrest, cardiac arrhythmia, respiratory failure, congenital heart disease, drowning/submersion) were felt to be high yield. Items with lower priority scores (toxic syndrome, meningitis/encephalitis) were felt by some physicians to not be necessary. Some clinical presentations (inborn error of metabolism, disseminated intravascular coagulation, adrenal disorders) were unexpected to be included in the curriculum and felt to be high yield by physicians

Twenty-one PEM physicians responded to the survey (88% response rate) with 20 (95%) respondents previously participating in PEMSOC CPD simulation activities. Four (19%) of respondents felt the curriculum included scenarios they did not feel were necessary, with three (14%) being unsure and the remaining 14 (67%) feeling all included scenarios were appropriate. Participant opinions on curriculum contents are detailed in Fig. 2: PEMSOC interim curriculum survey results regarding curriculum contents. Presentations the PEM physicians group felt should be included (but were not) were multi-system trauma (the subject of trauma not in the scope of this curriculum), pericardial tamponade (actually included in the curriculum), increased intracranial pressure (also included in the curriculum), traumatic arrest (not in the scope of this curriculum), violent/agitated patient, and the critical procedure of Burr hole.

**Fig. 3:**
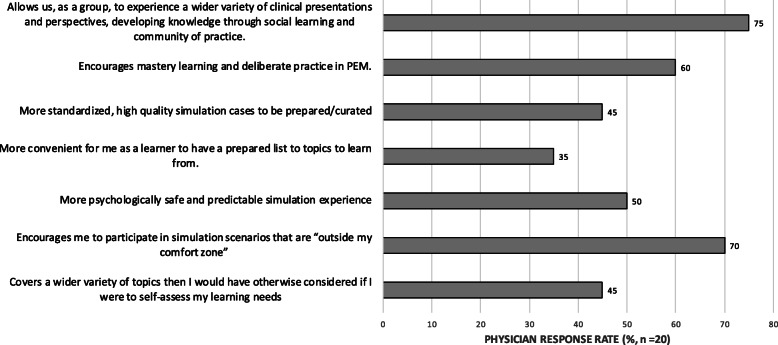
Physician opinions regarding the added value of CPD simulation over traditional ad hoc approach. (20/24 pediatric emergency medicine physicians responded to an online survey on personal opinions regarding simulations for continuing professional development adhering to a curriculum model, in comparison with simulations devised shortly in advance of a simulation session based on personal request (ad hoc approach). The majority of physicians felt the curriculum allowed them to experience more diverse clinical presentations outside of their comfort zone. The curriculum approach also encouraged mastery learning and created a more psychologically safe and predictable simulation experience.)

Regarding attitudes towards the PEM CPD simulation curriculum, 19 (90%) felt they could learn from a shared curriculum approach, with 19 (90%) feeling they could learn from our post-simulation, SimBITS knowledge dissemination package. Seventeen (81%) of physicians even felt our curriculum approach enhanced their learning compared to the previous, with the remainder being unsure (19%). No respondents felt unable to learn in our curriculum approach, and 11 (52%) respondents felt the curriculum would make it more likely they would participate in CPD simulation activities, with 8 (38%) being unsure and two (9.5%) responding no. PEM physician opinions on the CPD simulation curriculum are detailed in Fig. 3: Physician opinions regarding added value of CPD simulation over traditional ad hoc approach.

## Discussion

We describe a three-phase methodology for designing a simulation curriculum for CPD, whereby ranked data obtained from a systematic and deliberative approach is subsequently input into a project prioritization matrix to generate objective prioritization scores for use by program coordinators to plan, synthesize, and implement a final curriculum (Fig. 1: Continuing professional development simulation curriculum design process). To our knowledge, this is the first published methodology for curriculum design with CPD simulation activities.

Preliminary feedback from our curriculum approach was positive. The majority of PEM physicians found our curriculum approach educationally effective (potentially more than traditional ad hoc simulation). In addition, the vast majority of physicians found our curriculum comprehensive for their needs, with no participants disagreeing with clinical presentations included. Reassuringly, the clinical presentations our PEM physicians found to be of highest yield in the interim feedback survey were also the clinical presentations with the highest matrix prioritization scores (toxicology, inborn error of metabolism, cardiopulmonary arrest, cardiac arrhythmia, severe electrolyte abnormalities). Similarly, clinical presentations that PEM physicians felt were not required had lower matrix prioritization scores. There were also clinical presentations PEM physicians felt would be high yield but did not identify as important for curriculum inclusion, such as inborn error of metabolism, severe sepsis, and electrolyte abnormalities. These finding highlights and reaffirms a critical function of our curriculum approach: the capability to consider learning needs beyond those identified through the traditional self-directed approach.

Although our curriculum design process was derived for PEM subspecialty physicians, we believe our methodology is generalizable to other healthcare specialities interested in using simulation for CPD. Our phase 2 objectively incorporates the identified needs of multiple stakeholders involved with CPD, including participants, the healthcare system, and patients. Other healthcare specialities can adapt our prioritization matrix to suit their needs including incorporating differing clinical presentations and procedures, mathematical algorithms for calculating priority scores, and data categories to suit their needs. Alternative data categories for use in other priority matrices by other groups are shown in Table 4. We also intentionally designed our phase 3 of curriculum synthesis, implementation, and continuous review to rely on expert opinion of local education leaders. Our approach was applied to an acute care medical speciality in a fashion we felt best suited our local needs. However, our approach allows other healthcare specialities to select different numbers of clinical/presentations for curriculum inclusion, interpret prioritization score data to map different simulation curricula, implement their own CPD simulation programs compatible to their learning audience, and utilize variable knowledge dissemination techniques. While relying on local expert opinion creates more difficulty in consistently replicating curricula, we felt our approach was strengthened by the intrinsic conceptual flexibility of our methodology to accommodate the countless local needs, barriers, and variations of CPD simulation practice.

**Table 4:** Alternative data categories to consider with priority matrices in other CPD curriculum design processes

CPD domain	Alternative matrix data category			
Individual physician	Self-identified clinical presentations for physicians to practice via simulation^1^	Clinical presentations causing high anxiety to practicing physicians	Self-identified “interesting cases” for physicians	
Interprofessional/team	Interprofessional colleague (i.e., nursing, social work, respiratory therapist, pharmacy) identified priority topics^1^	Common learning needs identified from peer review processes	Priority cases requiring high level interprofessional collaboration (e.g., cardiopulmonary resuscitations with chest compression)	
Healthcare system	Administration/leadership needs^1^	Human resources and occupational health needs (e.g., personal protective equipment use)	Responding to emerging health threats (e.g., SARS/Ebola/terrorism/disaster)	
Patient and family-centered care	Priorities as identified by patient/family council committees	Priorities as identified from hospital patient experience committees	Code blue/pink/mortality review committee recommendations	Learning points from quality improvement and patient safety rounds^1^
Regulation/maintenance of certification program	Local governmental registry suggested priorities (e.g., provincial colleges of physicians and surgeons)	National certifying body priorities (e.g., Royal College of Physicians and Surgeons of Canada)^1^	High medico-legal risk priorities from malpractice data (e.g., Canadian Medical Protective Association)	
Simulation operation logistics	Feasibility of conducting simulation for learning objectives^1^	Value of performing simulation for learning compared to other learning methods^1^		

^1^Utilized by authors in the described matrix in this matrix

## Limitations

Unfortunately, due to the limitations of our single-center design, we determined a priori that we would be unable to address the above-proposed hypothesis comparing the educational effectiveness of our curricular approach to ad hoc SBME for CPD. It was not feasible in our single academic center to have some members of our pediatric emergency department in the curriculum group and others in a traditional stream. This is a major limitation of our current protocol requiring further investigation in future, multi-center, comparative studies. We do believe there is benefit to curricular integration with CPD simulation on learning outcomes, although additional studies regarding effect on learner knowledge, skills, and performance are required. Additional unique learning outcomes to examine from a CPD context include voluntary participation rates for CPD, economic analysis of an integrated simulation curriculum for CPD compared to ad hoc design, correlations with MOC certification performance and, of course, impact on real patient outcomes. Nonetheless, our protocol design process described here is the first step toward these future studies, which is critical, as SBME is increasingly utilized for CPD.

## Conclusions

We describe a novel three-phase process for curriculum design in simulation activities targeting independently practicing physicians for continuing professional development (CPD). The highlights of our approach are (1) an adaptable prioritization matrix capable of objectively ranking and identifying high-priority subjects to include using data collected from an in-depth perceived and unperceived needs analysis and (2) a knowledge gap sharing tool to facilitate group learning and community of practice amongst physicians. This methodology is valuable as simulation activities are increasingly embraced and required as CPD activities.

## Supplementary information

**Additional file 1.** Needs assessment survey.

**Additional file 2.** Sample simulation scenario.

**Additional file 3.** Sample SimBITS.

**Additional file 4.** Interim feedback survey.

## Data Availability

All the datasets used and/or analyzed during the current study are available from the corresponding author on reasonable request.
