# Supplementary material for: Development of an in situ simulation-based continuing professional development curriculum in pediatric emergency medicine
Source: Adv Simul (Lond). 2020 Jul 1;5:12. doi: 10.1186/s41077-020-00129-x (PMC7326623; doi:10.1186/s41077-020-00129-x)
Supplement: Supplementary file 1 — Additional file 1. Needs assessment survey. [file 41077_2020_129_MOESM1_ESM.pdf]

# Q1 Have you participated in simulations held in our ED (in situ simulations)? Select all that apply

Answered: 16 Skipped: 1

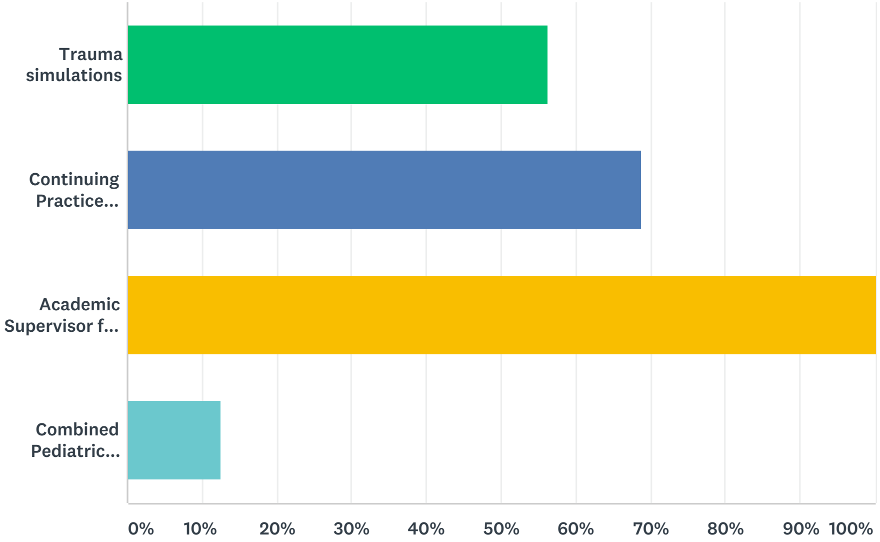


| ANSWER CHOICES | RESPONSES | |
|---|---|---|
| Trauma simulations | 56.25% | 9 |
| Continuing Practice (Staff-level) Simulation (***note that in this survey, this is the main type of simulation we are seeking feedback on) (held q4weeks) | 68.75% | 11 |
| Academic Supervisor for Resident Simulations | 100.00% | 16 |
| Combined Pediatric Emergency/NICU/Obs+Gyne Megacode Simulations (held 1-2 times a year) | 12.50% | 2 |
| Total Respondents: 16 | | |

# Q2 Did your professional training include regular simulation training (in situ or simulation center based) as part of its curriculum?

Answered: 17 Skipped: 0

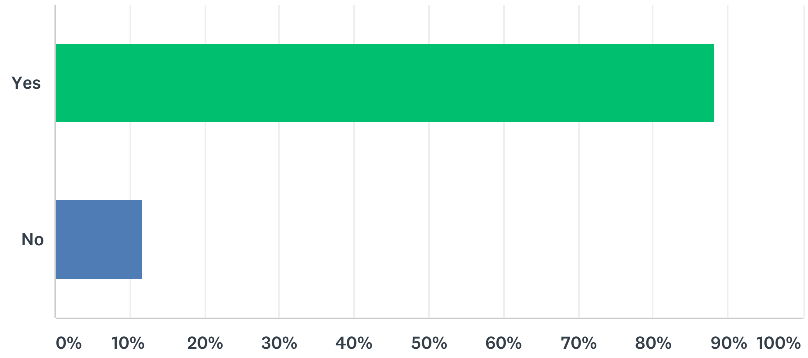


| ANSWER CHOICES | RESPONSES | |
|---|---|---|
| Yes | 88.24% | 15 |
| No | 11.76% | 2 |
| Total Respondents: 17 | | |

## Q3 How many total years have you been practicing/working in a Pediatric Emergency Setting? Include time not at McMaster. Do not include years of training in your total.

Answered: 17 Skipped: 0

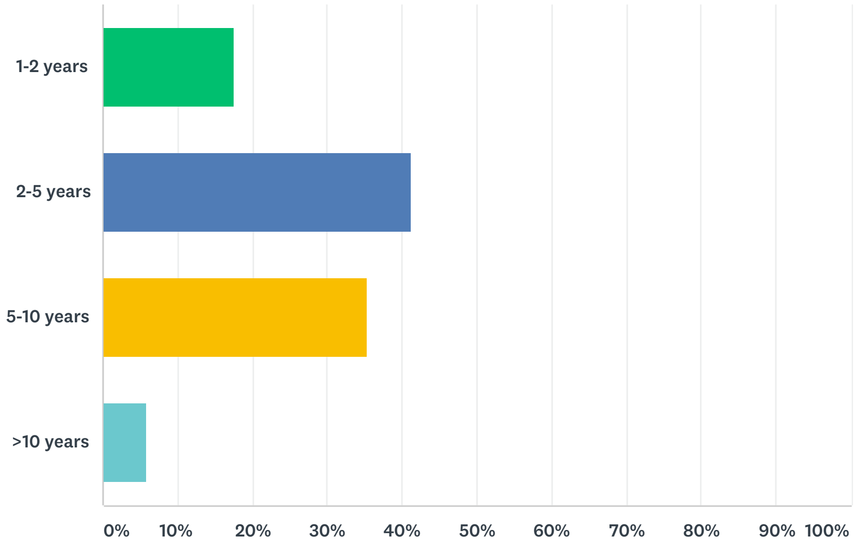


| ANSWER CHOICES | RESPONSES | |
|---|---|---|
| 1-2 years | 17.65% | 3 |
| 2-5 years | 41.18% | 7 |
| 5-10 years | 35.29% | 6 |
| >10 years | 5.88% | 1 |
| TOTAL | | 17 |

## Q4 What component of simulation contributes the most to your learning when you participate in our Continuing Practice Simulations?

Answered: 17 Skipped: 0

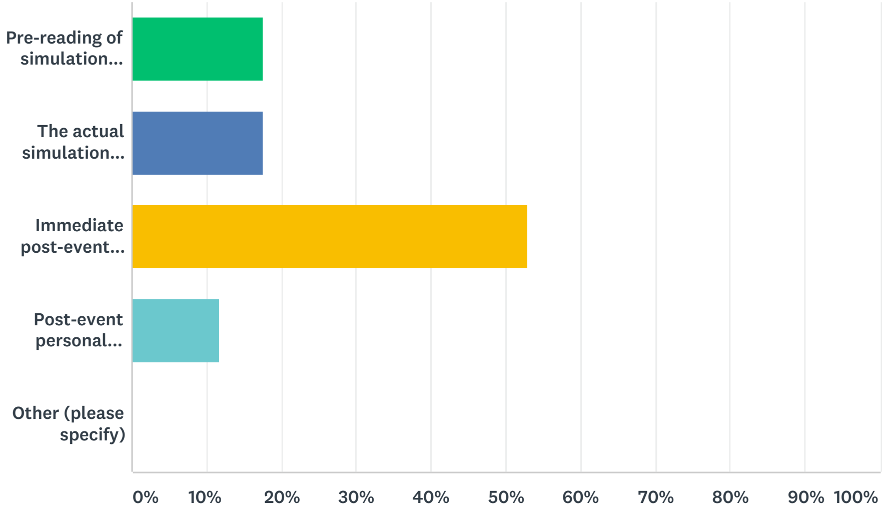


| ANSWER CHOICES | RESPONSES | |
|---|---|---|
| Pre-reading of simulation topic prior the simulation | 17.65% | 3 |
| The actual simulation event | 17.65% | 3 |
| Immediate post-event debriefing | 52.94% | 9 |
| Post-event personal reflection, and readings on personal time | 11.76% | 2 |
| Other (please specify) | 0.00% | 0 |
| TOTAL | | 17 |

## Q5 What are the three (3) most common resuscitations that you have encountered in actual clinical practice working in the McMaster Pediatric Emergency Department?

Answered: 17 Skipped: 0

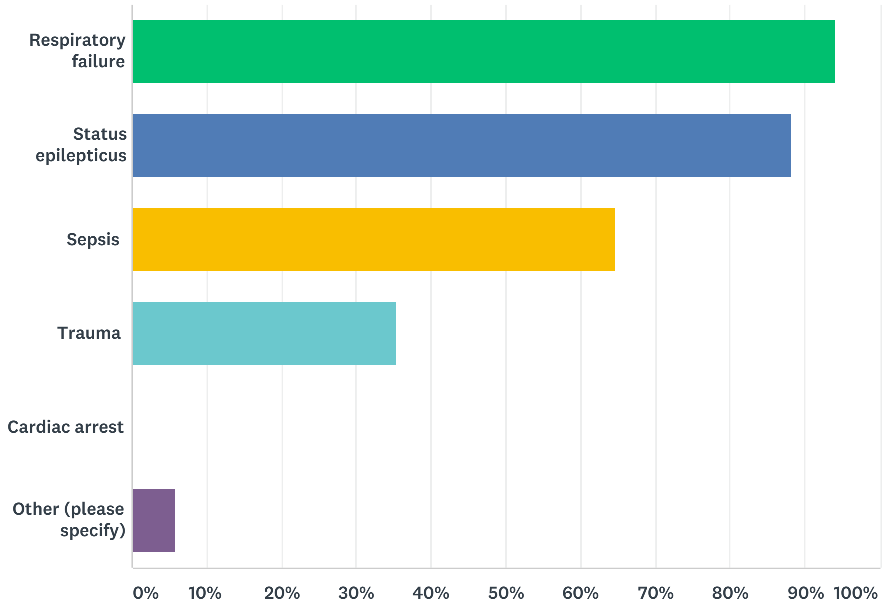


| ANSWER CHOICES | RESPONSES | |
|---|---|---|
| Respiratory failure | 94.12% | 16 |
| Status epilepticus | 88.24% | 15 |
| Sepsis | 64.71% | 11 |
| Trauma | 35.29% | 6 |
| Cardiac arrest | 0.00% | 0 |
| Other (please specify) | 5.88% | 1 |
| Total Respondents: 17 | | |

# Q6 Do you agree that it is important to cover rare but critical resuscitations (e.g. TCA overdose, difficult airway) in our simulations?

Answered: 17 Skipped: 0

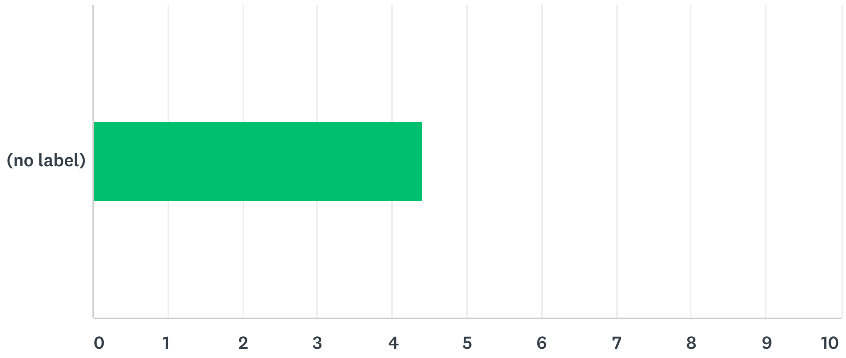


| | STRONGLY DISAGREE | DISAGREE | NEUTRAL | AGREE | STRONGLY AGREE | TOTAL | WEIGHTED AVERAGE |
|---|---|---|---|---|---|---|---|
| (no label) | 0.00%<br>0 | 0.00%<br>0 | 0.00%<br>0 | 58.82%<br>10 | 41.18%<br>7 | 17 | 4.41 |

# Q7 What objectives or skills would you like to work on in future Continuing Practice Simulations? Please rank your top five (5) choices, with 1 being most important.

Answered: 17 Skipped: 0

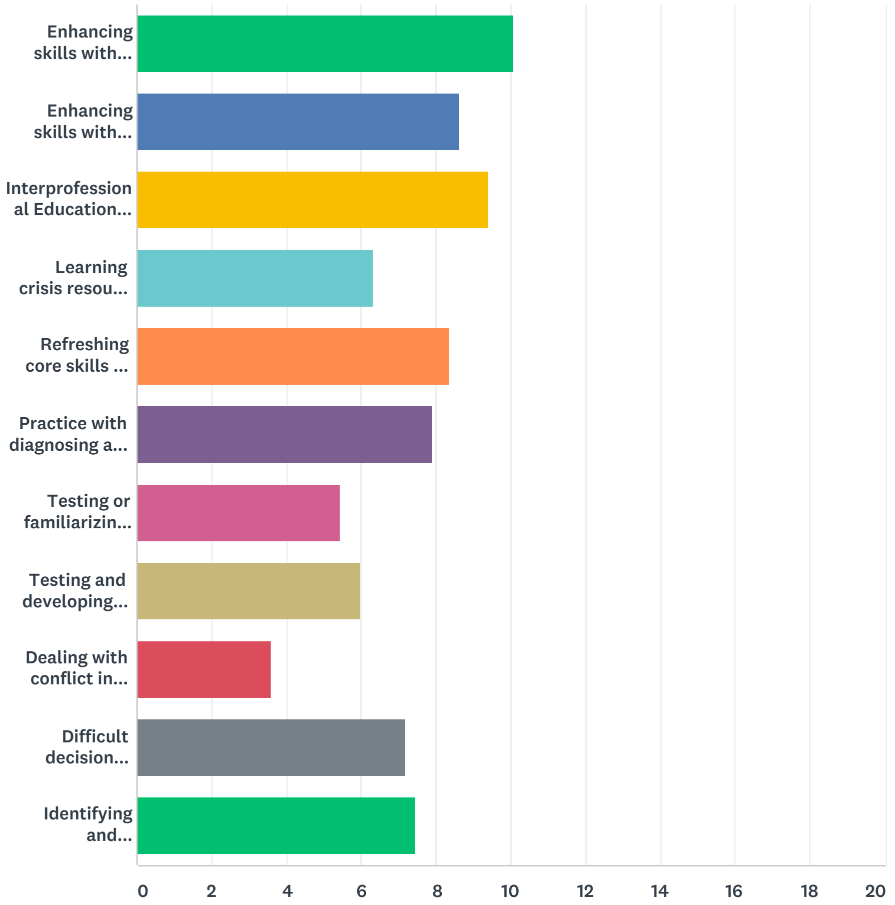


| | 1 | 2 | 3 | 4 | 5 | 6 | 7 | 8 | 9 | 10 | 11 | TOTAL |
|---|---|---|---|---|---|---|---|---|---|---|---|---|
| Enhancing skills with critical Pediatric Emergency procedures (e.g. chest tube insertion, intubation, casting) | 50.00% 7 | 21.43% 3 | 21.43% 3 | 0.00% 0 | 7.14% 1 | 0.00% 0 | 0.00% 0 | 0.00% 0 | 0.00% 0 | 0.00% 0 | 0.00% 0 | 14 |
| Enhancing skills with critical but rare Pediatric Emergency procedures (e.g. pericardiocentesis, burr holes, surgical airways) | 14.29% 2 | 28.57% 4 | 14.29% 2 | 21.43% 3 | 7.14% 1 | 7.14% 1 | 0.00% 0 | 7.14% 1 | 0.00% 0 | 0.00% 0 | 0.00% 0 | 14 |
| Interprofessional Education, Teamwork and Communication skills in crisis scenarios | 41.67% 5 | 16.67% 2 | 33.33% 4 | 0.00% 0 | 0.00% 0 | 0.00% 0 | 0.00% 0 | 0.00% 0 | 0.00% 0 | 8.33% 1 | 0.00% 0 | 12 |

| | | | | | | | | | | | | |
|---|---|---|---|---|---|---|---|---|---|---|---|---|
| Learning crisis resource management skills in resuscitations | 0.00% 0 | 0.00% 0 | 16.67% 1 | 33.33% 2 | 16.67% 1 | 0.00% 0 | 16.67% 1 | 0.00% 0 | 0.00% 0 | 0.00% 0 | 16.67% 1 | 6 |
| Refreshing core skills and algorithms in Pediatric Emergency care (i.e. PALS, NRP, CPR, Defibrillation) | 9.09% 1 | 27.27% 3 | 27.27% 3 | 18.18% 2 | 9.09% 1 | 0.00% 0 | 0.00% 0 | 0.00% 0 | 0.00% 0 | 0.00% 0 | 9.09% 1 | 11 |
| Practice with diagnosing and managing rare pediatric resuscitations. | 10.00% 1 | 10.00% 1 | 10.00% 1 | 10.00% 1 | 50.00% 5 | 10.00% 1 | 0.00% 0 | 0.00% 0 | 0.00% 0 | 0.00% 0 | 0.00% 0 | 10 |
| Testing or familiarizing yourself with new protocols, order sets or pathways | 0.00% 0 | 0.00% 0 | 0.00% 0 | 28.57% 2 | 14.29% 1 | 0.00% 0 | 28.57% 2 | 14.29% 1 | 0.00% 0 | 0.00% 0 | 14.29% 1 | 7 |
| Testing and developing quality improvement measures before implementing them into ED care. | 0.00% 0 | 0.00% 0 | 12.50% 1 | 25.00% 2 | 12.50% 1 | 0.00% 0 | 12.50% 1 | 25.00% 2 | 12.50% 1 | 0.00% 0 | 0.00% 0 | 8 |
| Dealing with conflict in acute care situations (interprofessional, or between patients/families and healthcare teams) | 0.00% 0 | 0.00% 0 | 0.00% 0 | 0.00% 0 | 20.00% 1 | 0.00% 0 | 0.00% 0 | 0.00% 0 | 60.00% 3 | 20.00% 1 | 0.00% 0 | 5 |
| Difficult decision making/diagnostic dilemmas during resuscitations | 0.00% 0 | 30.00% 3 | 10.00% 1 | 20.00% 2 | 10.00% 1 | 10.00% 1 | 0.00% 0 | 0.00% 0 | 0.00% 0 | 20.00% 2 | 0.00% 0 | 10 |
| Identifying and highlighting safety gaps | 9.09% 1 | 9.09% 1 | 9.09% 1 | 27.27% 3 | 27.27% 3 | 9.09% 1 | 0.00% 0 | 0.00% 0 | 0.00% 0 | 0.00% 0 | 9.09% 1 | 11 |

# Q8 Please select from the following list, clinical presentations where you feel there is an educational need to address with future Continuing Practice Simulations. Please select all that apply.(NB*** These options are from the 2013 Royal College of Physicians and Surgeons of Canada objectives for training in Pediatric Emergency Medicine)

Answered: 17 Skipped: 0

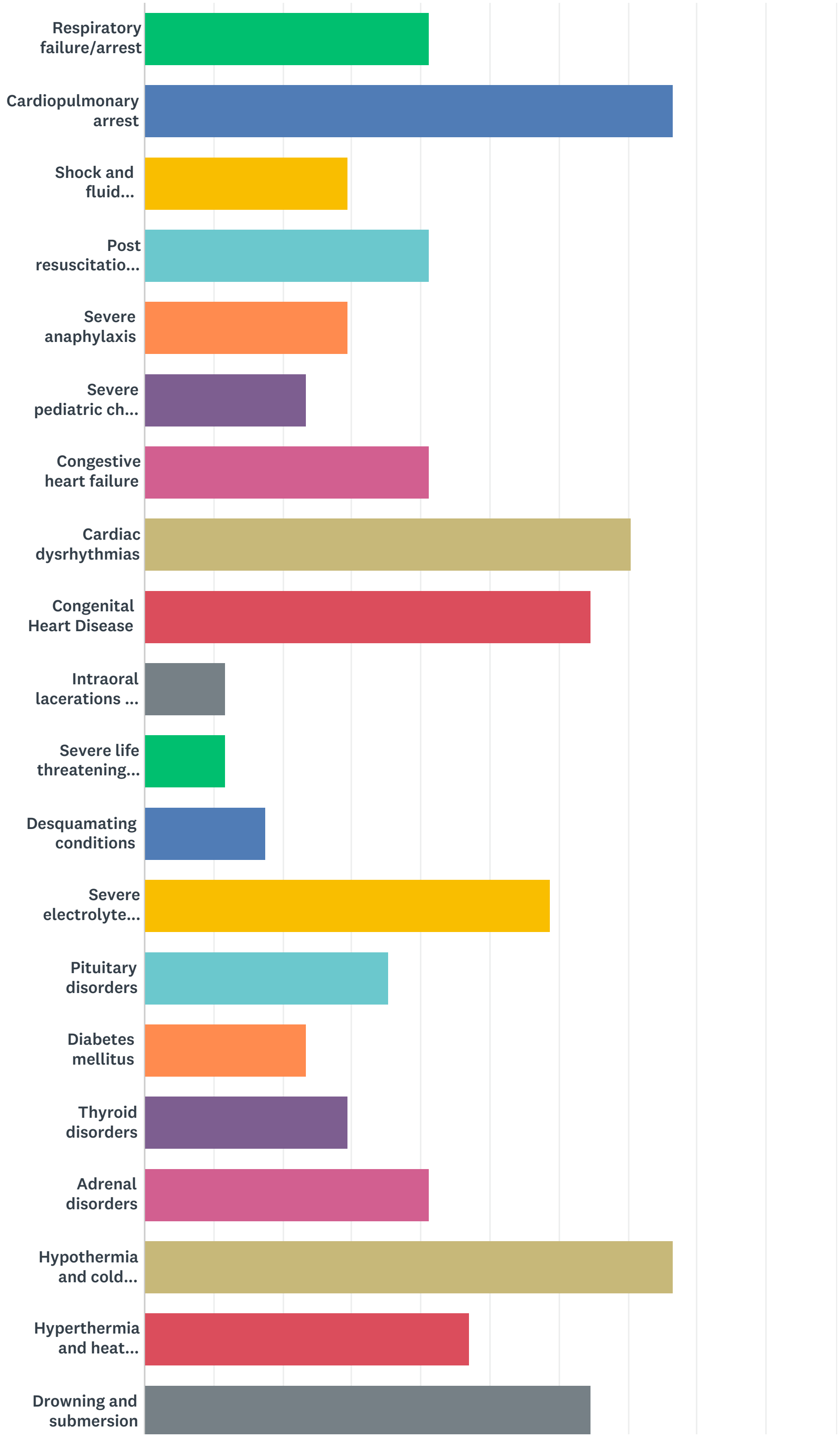

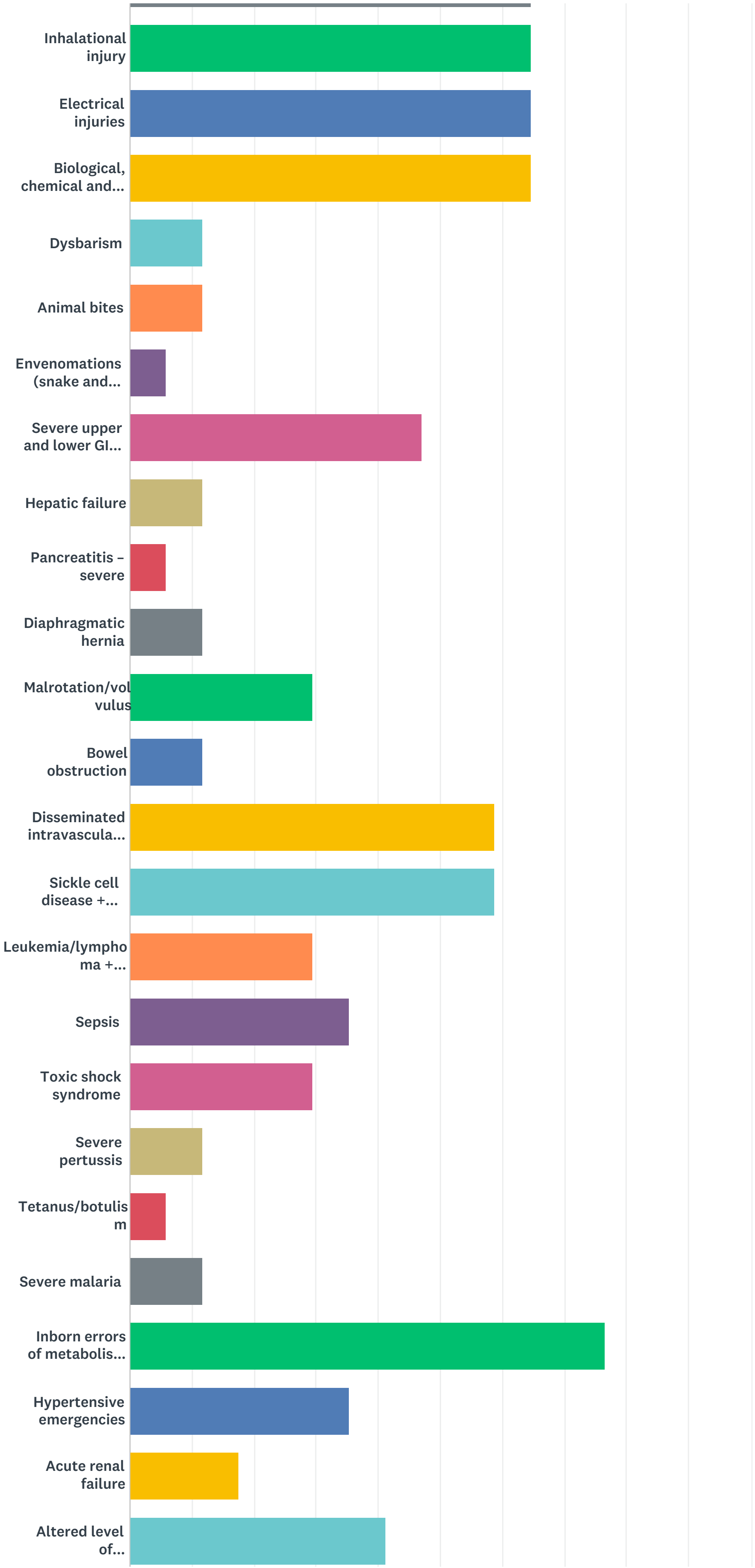
Inhalational injury
Electrical injuries
Biological, chemical and...
Dysbarism
Animal bites
Envenomations (snake and...
Severe upper and lower GI...
Hepatic failure
Pancreatitis – severe
Diaphragmatic hernia
Malrotation/volvulus
Bowel obstruction
Disseminated intravascula...
Sickle cell disease +...
Leukemia/lymphoma +...
Sepsis
Toxic shock syndrome
Severe pertussis
Tetanus/botulism
Severe malaria
Inborn errors of metabolis...
Hypertensive emergencies
Acute renal failure
Altered level of...

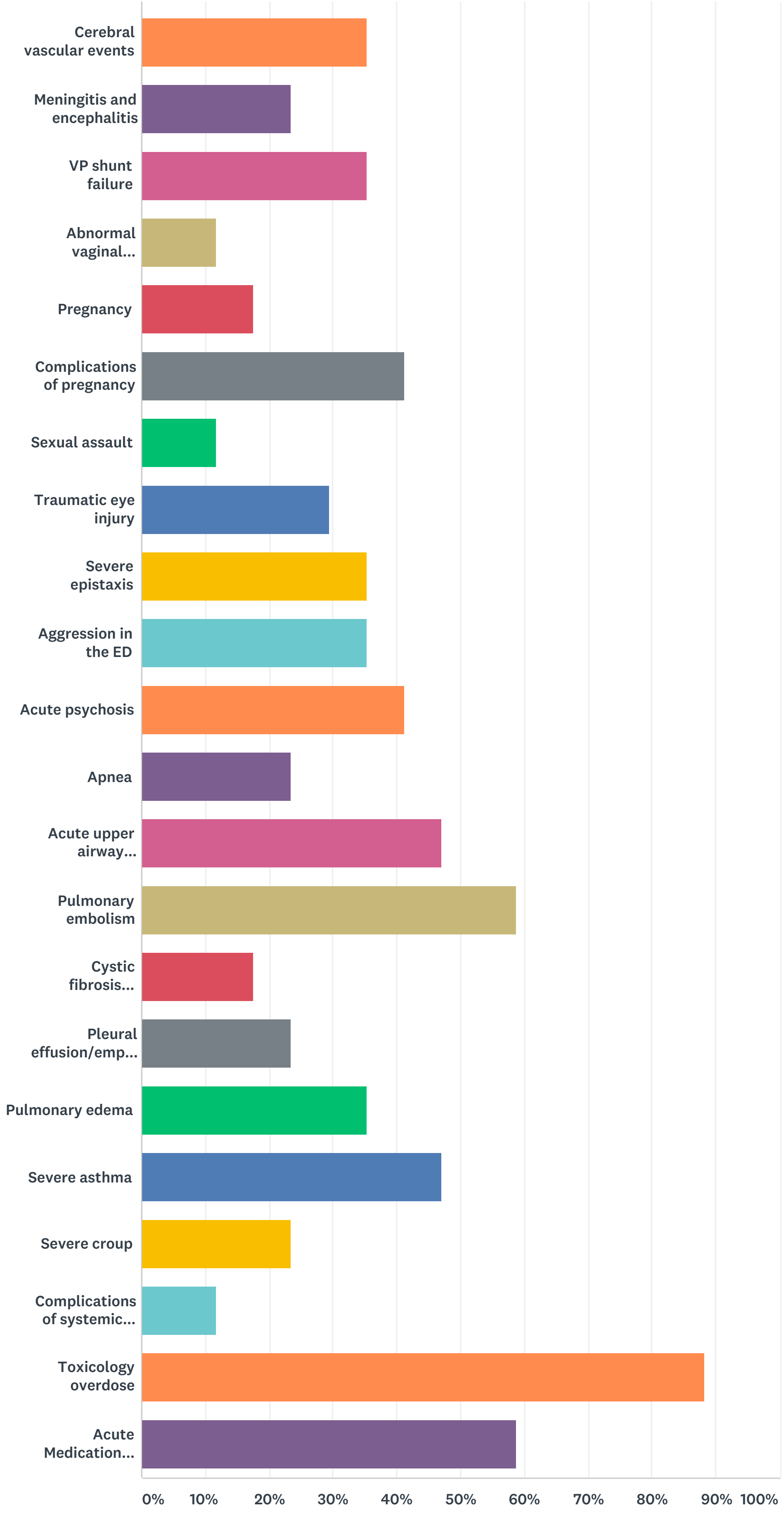


| ANSWER CHOICES | RESPONSES |
| --- | --- |

| | | |
|---|---|---|
| Respiratory failure/arrest | 41.18% | 7 |
| Cardiopulmonary arrest | 76.47% | 13 |
| Shock and fluid resuscitation | 29.41% | 5 |
| Post resuscitation management | 41.18% | 7 |
| Severe anaphylaxis | 29.41% | 5 |
| Severe pediatric chest pain | 23.53% | 4 |
| Congestive heart failure | 41.18% | 7 |
| Cardiac dysrhythmias | 70.59% | 12 |
| Congenital Heart Disease | 64.71% | 11 |
| Intraoral lacerations and soft tissue injuries | 11.76% | 2 |
| Severe life threatening rashes | 11.76% | 2 |
| Desquamating conditions | 17.65% | 3 |
| Severe electrolyte disturbances (sodium, potassium, calcium) | 58.82% | 10 |
| Pituitary disorders | 35.29% | 6 |
| Diabetes mellitus | 23.53% | 4 |
| Thyroid disorders | 29.41% | 5 |
| Adrenal disorders | 41.18% | 7 |
| Hypothermia and cold related injuries | 76.47% | 13 |
| Hyperthermia and heat related illnesses | 47.06% | 8 |
| Drowning and submersion | 64.71% | 11 |
| Inhalational injury | 64.71% | 11 |
| Electrical injuries | 64.71% | 11 |
| Biological, chemical and radiation exposures | 64.71% | 11 |
| Dysbarism | 11.76% | 2 |
| Animal bites | 11.76% | 2 |
| Envenomations (snake and marine) | 5.88% | 1 |
| Severe upper and lower GI bleed | 47.06% | 8 |
| Hepatic failure | 11.76% | 2 |
| Pancreatitis – severe | 5.88% | 1 |
| Diaphragmatic hernia | 11.76% | 2 |
| Malrotation/volvulus | 29.41% | 5 |
| Bowel obstruction | 11.76% | 2 |
| Disseminated intravascular coagulation | 58.82% | 10 |
| Sickle cell disease + complications | 58.82% | 10 |
| Leukemia/lymphoma + complications | 29.41% | 5 |
| Sepsis | 35.29% | 6 |
| Toxic shock syndrome | 29.41% | 5 |
| Severe pertussis | 11.76% | 2 |
| Tetanus/botulism | 5.88% | 1 |
| Severe malaria | 11.76% | 2 |

| | | |
|---|---|---|
| Inborn errors of metabolism and metabolic catastrophe | 76.47% | 13 |
| Hypertensive emergencies | 35.29% | 6 |
| Acute renal failure | 17.65% | 3 |
| Altered level of consciousness | 41.18% | 7 |
| Cerebral vascular events | 35.29% | 6 |
| Meningitis and encephalitis | 23.53% | 4 |
| VP shunt failure | 35.29% | 6 |
| Abnormal vaginal bleeding | 11.76% | 2 |
| Pregnancy | 17.65% | 3 |
| Complications of pregnancy | 41.18% | 7 |
| Sexual assault | 11.76% | 2 |
| Traumatic eye injury | 29.41% | 5 |
| Severe epistaxis | 35.29% | 6 |
| Aggression in the ED | 35.29% | 6 |
| Acute psychosis | 41.18% | 7 |
| Apnea | 23.53% | 4 |
| Acute upper airway obstruction | 47.06% | 8 |
| Pulmonary embolism | 58.82% | 10 |
| Cystic fibrosis exacerbations | 17.65% | 3 |
| Pleural effusion/empyema | 23.53% | 4 |
| Pulmonary edema | 35.29% | 6 |
| Severe asthma | 47.06% | 8 |
| Severe croup | 23.53% | 4 |
| Complications of systemic lupus erythematosis/rheumatoid arthritis | 11.76% | 2 |
| Toxicology overdose | 88.24% | 15 |
| Acute Medication Withdrawal | 58.82% | 10 |
| Total Respondents: 17 | | |

# Q9 Please indicate what critical resuscitative procedures you feel like you wish to address in future Continuing Practice Simulations. Select all that apply.(NB***Options from this list are a selected from procedural skills expected from the 2013 Royal College of Physicians and Surgeons of Canada objectives for training in Pediatric Emergency Medicine)

Answered: 17 Skipped: 0

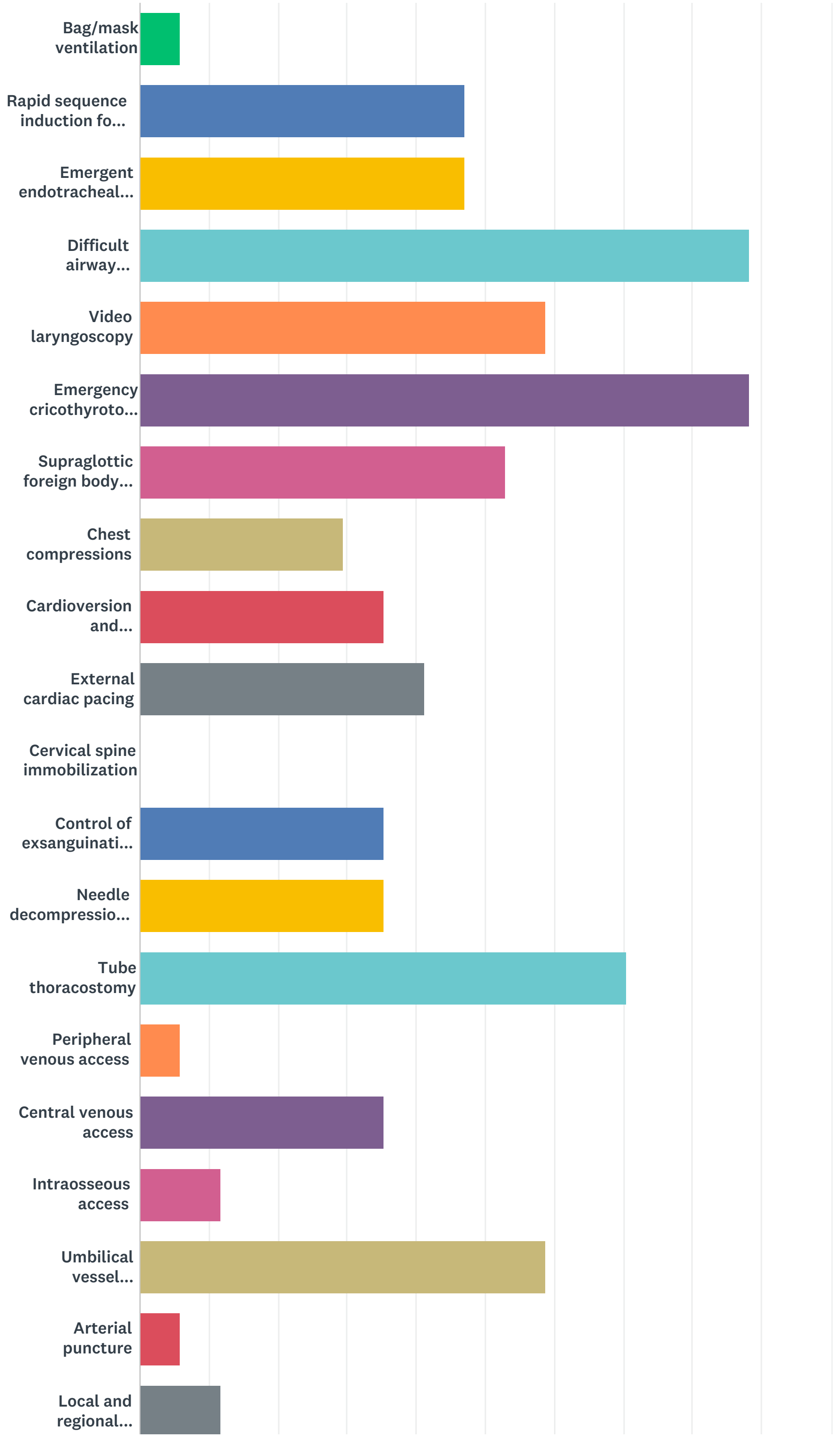

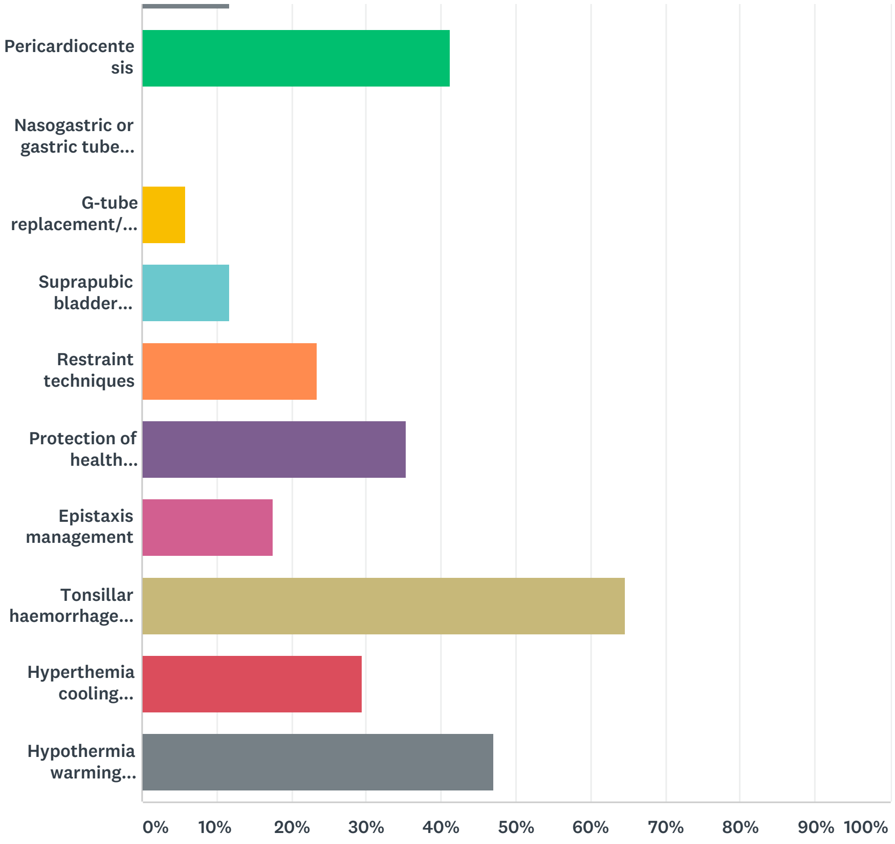


| ANSWER CHOICES | RESPONSES | |
|---|---|---|
| Bag/mask ventilation | 5.88% | 1 |
| Rapid sequence induction for intubation | 47.06% | 8 |
| Emergent endotracheal intubation | 47.06% | 8 |
| Difficult airway management | 88.24% | 15 |
| Video laryngoscopy | 58.82% | 10 |
| Emergency cricothyrotomy and transtracheal ventilation | 88.24% | 15 |
| Supraglottic foreign body removal | 52.94% | 9 |
| Chest compressions | 29.41% | 5 |
| Cardioversion and defibrillation | 35.29% | 6 |
| External cardiac pacing | 41.18% | 7 |
| Cervical spine immobilization | 0.00% | 0 |
| Control of exsanguinating external haemorrhage | 35.29% | 6 |
| Needle decompression of chest | 35.29% | 6 |
| Tube thoracostomy | 70.59% | 12 |
| Peripheral venous access | 5.88% | 1 |
| Central venous access | 35.29% | 6 |
| Intraosseous access | 11.76% | 2 |
| Umbilical vessel catherization | 58.82% | 10 |
| Arterial puncture | 5.88% | 1 |
| Local and regional anesthesia | 11.76% | 2 |

| | | |
|---|---|---|
| Pericardiocentesis | 41.18% | 7 |
| Nasogastric or gastric tube insertion | 0.00% | 0 |
| G-tube replacement/access | 5.88% | 1 |
| Suprapubic bladder catherization | 11.76% | 2 |
| Restraint techniques | 23.53% | 4 |
| Protection of health professionals from hazardous exposures | 35.29% | 6 |
| Epistaxis management | 17.65% | 3 |
| Tonsillar haemorrhage management | 64.71% | 11 |
| Hyperthemia cooling procedures | 29.41% | 5 |
| Hypothermia warming procedures | 47.06% | 8 |
| Total Respondents: 17 | | |

## Q10 Please indicate any additional topics, scenarios or procedures you would like to cover that were not included in prior questions

Answered: 3 Skipped: 14

# Q11 Psychological safety (Part 1 of 2). We recognize simulation can be a threatening and anxiety producing experience for any participant, regardless of background. Above all, we are committed to protecting your psychological safety. Knowing that stress can never be fully eliminated from simulation, please select from the following actions we have previously done and should continue doing to make simulation more psychologically safe. Select your top choice.

Answered: 17 Skipped: 0

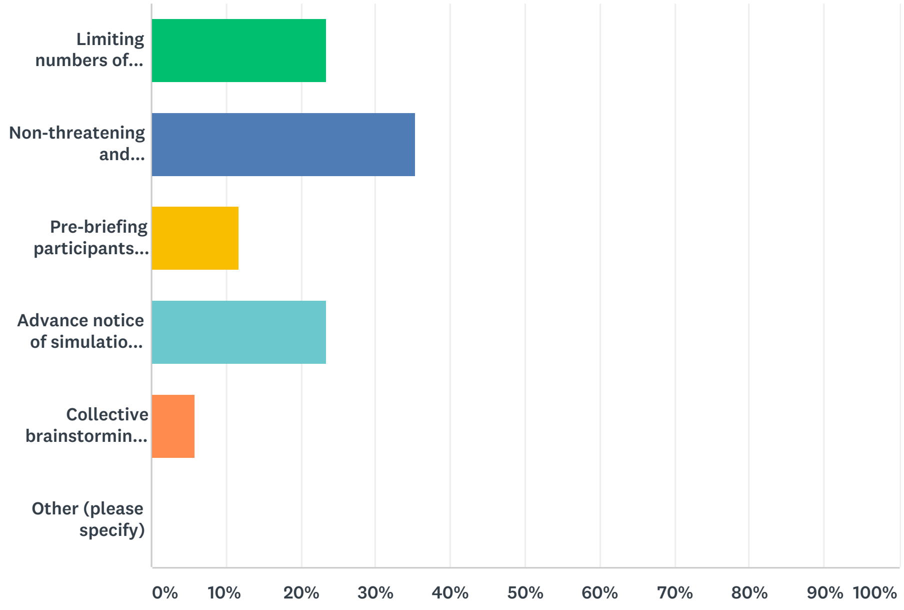


| ANSWER CHOICES | RESPONSES | |
|---|---|---|
| Limiting numbers of observers in a simulation | 23.53% | 4 |
| Non-threatening and non-patronizing debriefing style | 35.29% | 6 |
| Pre-briefing participants to set up of simulation and overarching objectives of the simulation before the event occurs (i.e. introduction to room, mannequin and facilitators) | 11.76% | 2 |
| Advance notice of simulation topics | 23.53% | 4 |
| Collective brainstorming of solutions to problems/concerns raised by simulation activities | 5.88% | 1 |
| Other (please specify) | 0.00% | 0 |
| TOTAL | | 17 |

# Q12 Psychological safety (Part 2 of 2). What do you feel is most anxiety provoking about in situ simulation? Select all that apply

Answered: 17 Skipped: 0

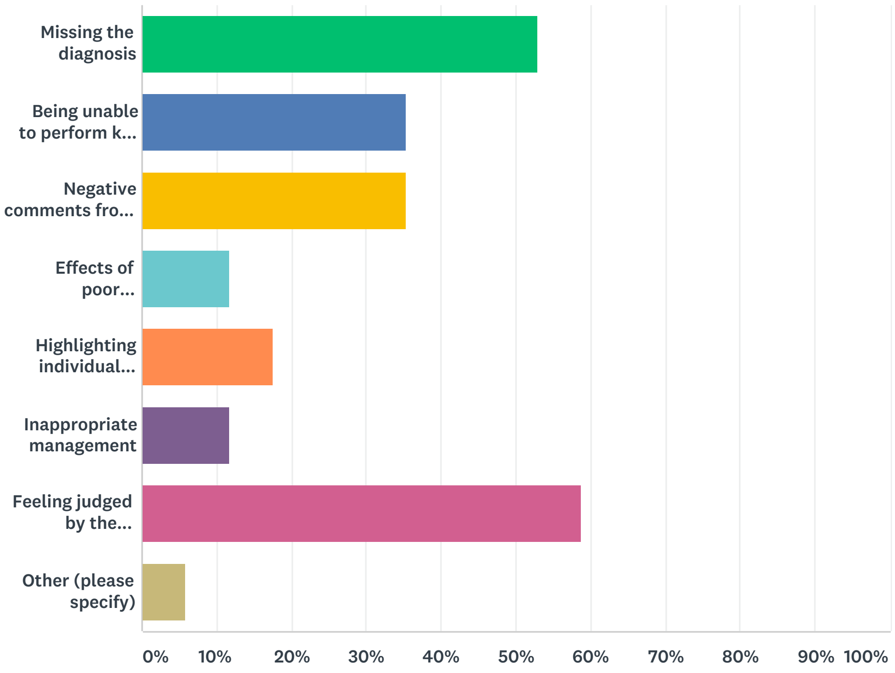


| ANSWER CHOICES | RESPONSES | |
|---|---|---|
| Missing the diagnosis | 52.94% | 9 |
| Being unable to perform key critical tasks | 35.29% | 6 |
| Negative comments from peers and interprofessional colleagues | 35.29% | 6 |
| Effects of poor performance on employment status | 11.76% | 2 |
| Highlighting individual performance gaps | 17.65% | 3 |
| Inappropriate management | 11.76% | 2 |
| Feeling judged by the simulation team | 58.82% | 10 |
| Other (please specify) | 5.88% | 1 |
| Total Respondents: 17 | | |

# Q13 Agree or disagree? Advance notice of simulation topics (Part 1 of 2). If it does not interfere with the simulation objectives, participants should be made aware of the general topic of simulation a minimum of 24h before the exercise

Answered: 17 Skipped: 0

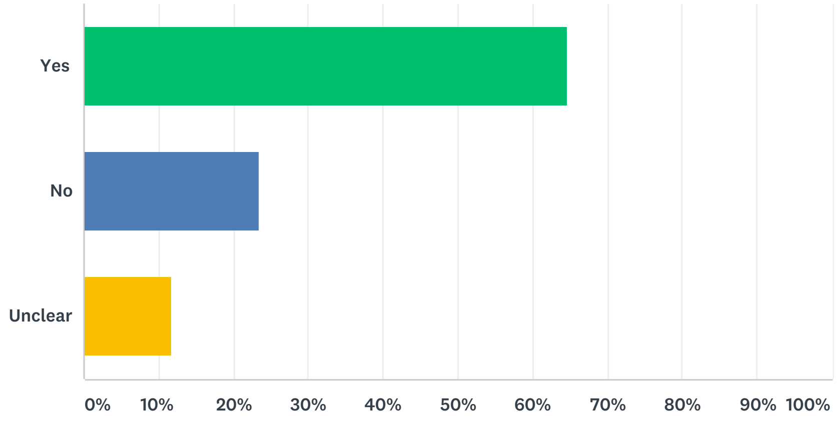


| ANSWER CHOICES | RESPONSES | |
|---|---|---|
| Yes | 64.71% | 11 |
| No | 23.53% | 4 |
| Unclear | 11.76% | 2 |
| Total Respondents: 17 | | |

# Q14 Agree or disagree? Advance notice of simulation topics (Part 2 of 2). If an objective of the simulation is to test system response and quality improvement, participants should not be made of aware of the general topic of simulation in advance.

Answered: 17 Skipped: 0

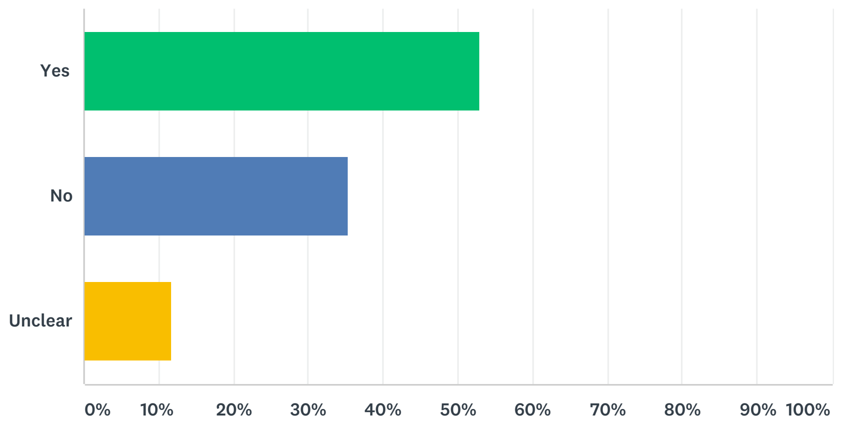


| ANSWER CHOICES | RESPONSES | |
|---|---|---|
| Yes | 52.94% | 9 |
| No | 35.29% | 6 |
| Unclear | 11.76% | 2 |
| Total Respondents: 17 | | |

## Q15 We recognize there are occasions where scheduled in situ simulations need to be postponed/cancelled (especially in a busy ED). Balancing that simulations are costly, and resource intensive to hold, please comment on 1-3 situations where you feel a scheduled in situ simulation activities should ED be postponed/cancelled.

Answered: 14 Skipped: 3

## Q16 Would you be interested in joining our simulation team to help facilitate and develop future simulation scenarios for either our own department or as part of an outreach team?  Please send an email to jamessleung@gmail.com if you are interested and we will be happy to chat!

Answered: 16 Skipped: 1

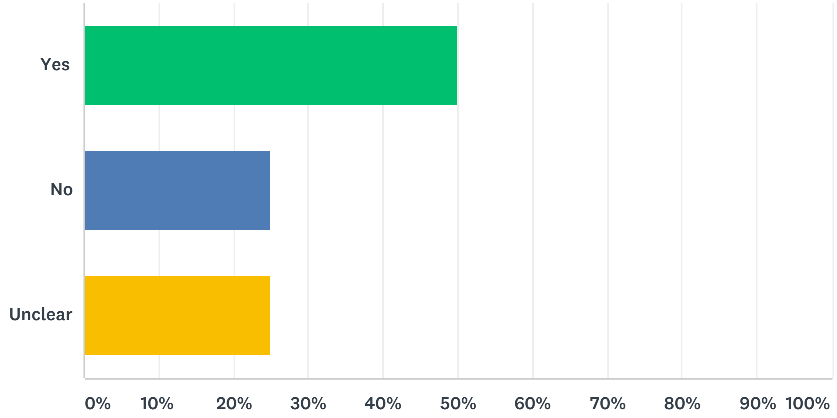


| ANSWER CHOICES | RESPONSES | |
|---|---|---|
| Yes | 50.00% | 8 |
| No | 25.00% | 4 |
| Unclear | 25.00% | 4 |
| Total Respondents: 16 | | |

# Q17 Our Simulation team publishes a monthly newsletter highlighting knowledge points gained from our simulation activities, with the objective of reaching a broader audience than the participants who had the benefit of participating in simulation, and improving our learning. Have you previously read our SimBits newsletter?

Answered: 17 Skipped: 0

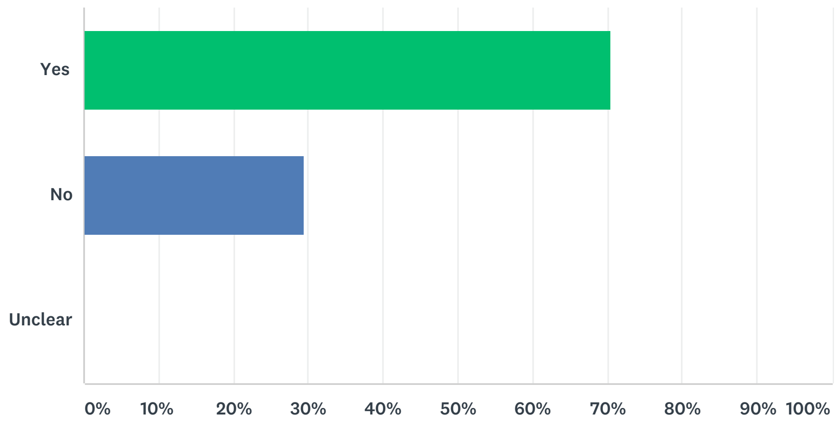


| ANSWER CHOICES | RESPONSES | |
|---|---|---|
| Yes | 70.59% | 12 |
| No | 29.41% | 5 |
| Unclear | 0.00% | 0 |
| TOTAL | | 17 |

# Q18 Does SimBits help your Continuing Professional Education if you did not actually participate in the simulation?

Answered: 17 Skipped: 0

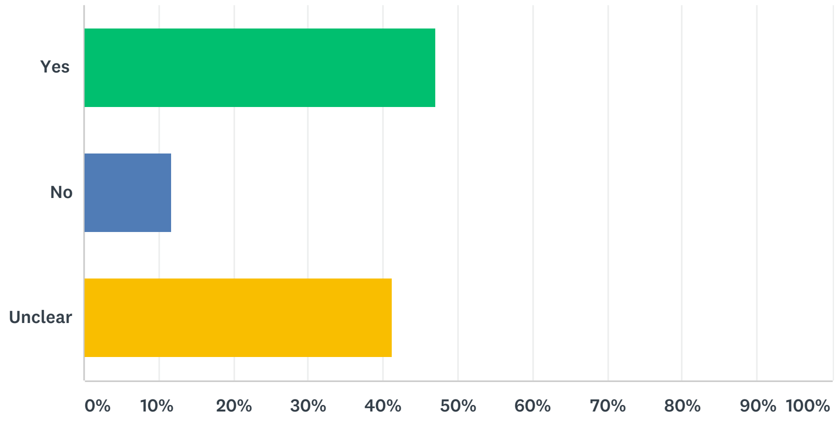


| ANSWER CHOICES | RESPONSES | |
|---|---|---|
| Yes | 47.06% | 8 |
| No | 11.76% | 2 |
| Unclear | 41.18% | 7 |
| TOTAL | | 17 |

## Q19 Do you feel threatened or unsafe from the content that is published in SimBits if you participated in the scenario?

Answered: 16 Skipped: 1

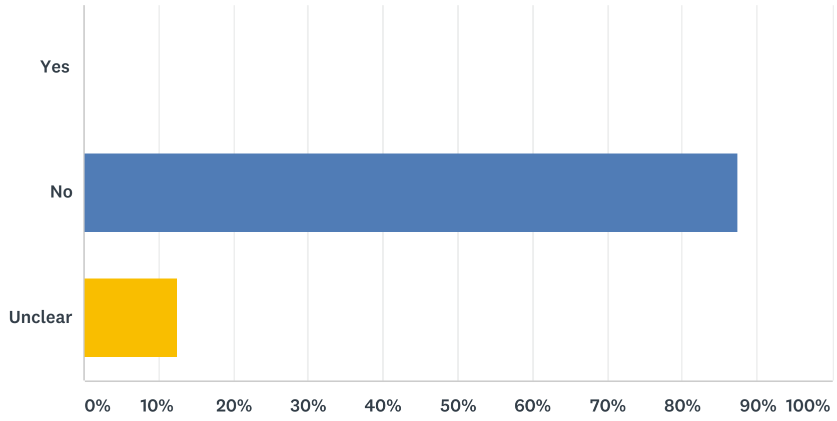


| ANSWER CHOICES | RESPONSES | |
|---|---|---|
| Yes | 0.00% | 0 |
| No | 87.50% | 14 |
| Unclear | 12.50% | 2 |
| TOTAL | | 16 |

# Q20 Please indicate barriers to reading SimBits. Select all that apply

Answered: 14 Skipped: 3

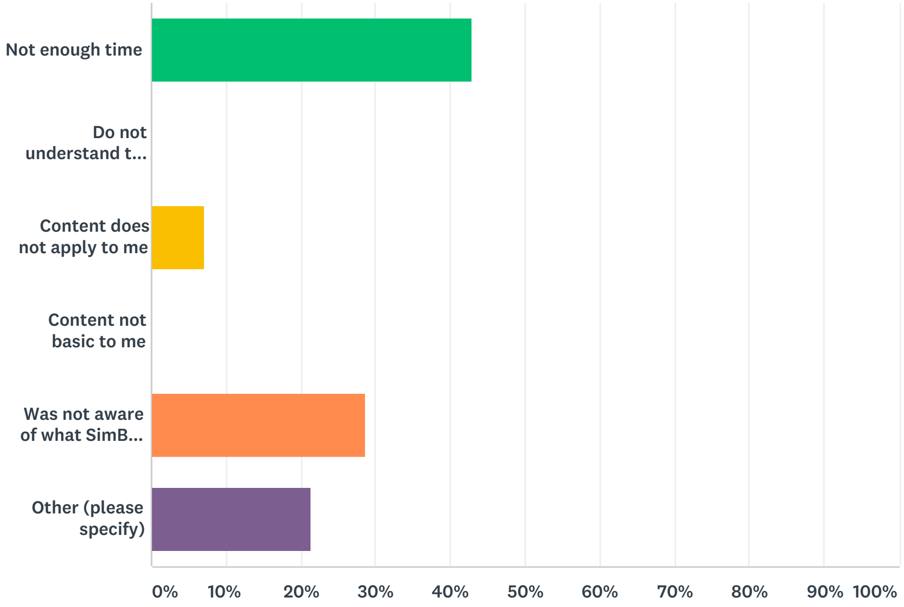


| ANSWER CHOICES | RESPONSES | |
|---|---|---|
| Not enough time | 42.86% | 6 |
| Do not understand the content | 0.00% | 0 |
| Content does not apply to me | 7.14% | 1 |
| Content not basic to me | 0.00% | 0 |
| Was not aware of what SimBits is | 28.57% | 4 |
| Other (please specify) | 21.43% | 3 |
| Total Respondents: 14 | | |
