# Supplementary material for: Development of an in situ simulation-based continuing professional development curriculum in pediatric emergency medicine
Source: Adv Simul (Lond). 2020 Jul 1;5:12. doi: 10.1186/s41077-020-00129-x (PMC7326623; doi:10.1186/s41077-020-00129-x)
Supplement: Supplementary file 2 — Additional file 2. Sample simulation scenario. [file 41077_2020_129_MOESM2_ESM.docx]

Continuing Practice In Situ Simulation 3 – TCA overdose

**Case ID Number: Continuing Practice 3**

**Author:**

James Leung

**Revising Authors:** Mohamed Eltorki, Mandeep Brar, Leanne Patel

**Date of Case Creation:** December 5, 2017 (Adapted from Kidsim SD0022)

**Date of last Case Revision Date:** April 4, 2019

**Target Audience:**

Interprofessional Acute Care Teams

- MD (FRCPC EM/PEM)
- RN
- Respiratory Therapy
- Pharmacist/Pharmacy Technician
- Diagnostic imaging (if available)
- PICU physician (if available)
- Social Work (if available)

**Simulation Difficulty Level:**

Advanced

**Objectives:**

By the end of the session, participants will:

*Knowledge:*

1. Recognize signs of a potential overdose
2. Management of agitated patient in the ED.
3. Recognize signs of a TCA overdose and pathophysiology of TCA overdose
4. Recognize ECG changes associated with severe TCA overdose and appropriate sodium bicarbonate management
5. Pathophysiology of TCA overdose
6. Complications and pathophysiology of TCA overdose – hypotension and seizure and appropriate management in an overdose patient
7. Indications for securing airway in a patient with altered LOC
8. Approach managing pediatric poisoning using the ABCDEF algorithm of toxicology: Airway, Breathing, Circulation, Decontamination, Elimination, Find antidote.
9. Metabolic side effects of bicarbonate and increasing hypercarbia secondary to increased CO2 production

*Skills*:

1. Adhere to logistical protocols with critical patient fan out, including calling for help/PICU support/activation of ED Code Blue System
2. Early consultation process with Poison Control.
3. Demonstrate use of appropriate bedside references such as Pediatric Broselow kits
4. Accurate recording in a crisis resource limited scenario.
5. Demonstrate safe medication administration practices, particularly selection, dosing and delivery of:
   1. Sodium bicarbonate bolus
   2. Intralipid rescue
6. Recognize value of repeated ECG in patient with TCA toxicity.

*Manager/Collaborator:*

1. Situational awareness and recognition of cardiogenic shock in a newborn
2. Succinctly collect, review and summarize information presented to teams during handover (EMS)
3. Effective closed loop communication to team members
4. Focused and systemic consultation practices adhering to SBAR practices
5. Team leader demonstrate inclusive leadership
6. Development of a shared mental model
7. Complete documentation of event
8. Demonstrate skill with crisis resource management particularly with:
   1. Role delegation
   2. Prioritization of orders/tests/medication administration

**Materials:**

*Location*: Emergency Department Resuscitation Bay

*Mannequin:* **Adult Manniquen*****

*Monitors:*

- Cardiorespiratory monitor including oxygen saturation monitor

*Physical Props / Equipment:*

- **Fake suicide note**
- Defibrillator + pediatric pads
- Oxygen delivery devices
  - low flow (nasal prongs, mask)
- Standard Airway Equipment
  - BVM
  - OPA / NPA
  - Laryngoscope with curved and straight blades
  - ETT sizes 5-7
  - CO2 detection device
  - Bougie or other rescue device
- Standard IV Equipment
  - IV catheters
  - IV Pump
  - IV tubing
- Advanced IV Equipment
  - IO gun
  - Three-way stop-cock push pull system
  - IO model for insertion of IV
- IV fluids:
  - NS
  - D5NS
  - IV pump/level 1 infuser/pressure bags
- Gastric lavage kit
- Medications
  - **Sodium bicarbonate**
  - **Intralipid emulsification**
  - **Activated charcoal**
  - Standard PALS medications
  - Sedation Medications
    - Lorazepam
    - Haldol
    - Olanzepine
  - RSI Kit
    - Etomidate
    - Succinocholyine
    - Rocuronium
    - Ketamine
    - Propofol
    - Fentanyl
    - Midazolam
  - Vasopressors
    - Dopamine
    - Dobutamine
    - Epinephrine
    - Norepinephrine
    - Milrinone
  - Tylenol
  - Fentanyl
  - Medication references/policies
  - Copy of HHS Pediatric Resuscitation Order Sheet

*Multi-Media:*

- Chest x-ray 1 (normal)
- EKG 1 (sinus tachycardia, first degree AV block , wide complex tachycardia, RSR’ (positive in avR) – initial ECG)
- EKG 2 (Worsening ECG – worsening wide complex QRS tachycardia)
- EKG 3 (Post treatment with bicarb)
- Initial labs – critical blood gas – severe metabolic acidosis, wide anion gap
  - - Follow-up gas post bicarb

*Confederates*:

- Parent
- PICU
- Toxicology/Poison Control

**LABORATORY SERVICES**

**COLLECTION SITE: NAME:**

**PATIENT ID:**

**PHYSICIAN:**

**RESULT REFERENCE RANGE**

**BLOOD GAS (CRITICAL VENOUS)**

**BLOOD GAS VALUES**

pH **7.2 C**** 7.35-7.45

pCO2 **47 mmHg** **H** 35-45

pO2 39 mmHg 35-45

cHCO3 **15 mmol/L** **L**  18-26

**OXIMETRY VALUES**

**ctHb 125 g/L H** 125-180

FO2Hb 98 % 88-100

FCOHb 0 % 0-1.5

fMetHb 2 % 0-1.5

**ELECTROLYTE VALUES**

cNa 141 mmol/L (138-148)

cK 4.6 mmol/L (3.6-5.6)

cCl 100 mmol/L (98-110)

cCa 1.5 mmol/L (0-2)

**METABOLIC VALUES**

cGlu 6 mmol/L (3.9-6.1)

cLactate **2.6 mmol/L H** (0-2)

**LABORATORY SERVICES**

**COLLECTION SITE: NAME:**

**PATIENT ID:**

**PHYSICIAN:**

**RESULT REFERENCE RANGE**

**BLOOD GAS (CRITICAL VENOUS)**

**BLOOD GAS VALUES**

pH 7.4 7.35-7.45

pCO2 **46 mmHg** **H** 35-45

pO2 39 mmHg 35-45

cHCO3 26 mmol/L 18-26

**OXIMETRY VALUES**

**ctHb 125 g/L H** 125-180

FO2Hb 98 % 88-100

FCOHb 0 % 0-1.5

fMetHb 2 % 0-1.5

**ELECTROLYTE VALUES**

cNa 141 mmol/L (138-148)

cK 4.6 mmol/L (3.6-5.6)

cCl 100 mmol/L (98-110)

cCa 1.5 mmol/L (0-2)

**METABOLIC VALUES**

cGlu 6 mmol/L (3.9-6.1)

cLactate **2.2 mmol/L H** (0-2)

**SCENARIO: (Duration 20-25 minutes simulation + 30-45min debrief)**

**Introduction (provided to participants):**

- **Location: Emergency Department Resuscitation Bay**
- EMS are transporting an agitated 16 year old male who took an overdose of an unknown medication. Found with a suicide note. History of anxiety and depression. No prior attempts. Parents last seen at breakfast at 0630h where he was well and healthy. Found by parents confused, and agitated. Parents are enroute.
- Initial vital signs: HR 130 | BP 112/80| RR 22 | SpO2 98%

**Case Progression:**

| **Scenario Transitions / Evolution** | **Effective Management** | **Ineffective Management** | **Notes** |
| --- | --- | --- | --- |
| 1. Assessment of toxic patient – agitated and uncooperative.  T: 38.5C PO/Ax  Weight 70kg  HR: 130 BP: 110/83, Sp02: 98% room air RR: 25  Blood glucose = 6  A: Patient. Moaning. No meningismus.  B: Air entry bilaterally, with crackles to bases. No wheeze.  C: CRT<2s. Flushed  D: GCS E4V4M4(GCS12). Confused, moaning to pain, moves/responsive to pain, eyes open. Normal tone.  E: Pupils 7mm and reactive to light. Dry mucous membranes. No rash  If palpated, will have no hepatospenomegaly, soft nontender abdomen. +++ active bowel sounds.  No smell of ETOH. No kussmaul’s breathing | - Call for help/support - **Places patient on monitors** - **EKG ordered – recognize potential TCA toxicity** - Recognizes anticholinergic toxidrome - Primary survey completed. Broselow tape - **Obtain access. Cap sugar**   **Critical VBG/cross and type/ED trauma requested (if possible)**   - **Consider charcoal** - Primary and secondary survey completed - no trauma identified - **Considers sedation for agitation** | - If no restraint applied , will be unable to get IV access. Patient will rip out IVs | - **Consider charcoal – may not want to give as confused any may vomit** - Combative. Will fight off oxygen if applied. Needs to be held down for IVs - **Move to phase 2 with administration of sedation medication or 5 minutes elapse** |
| 2. Decreased level of consciousness and poor perfusion (wide complex tachycardia)  37.8C \| BP: 80/63 \| HR 150 \| SpO2 85% \| RR 25  A. Unchanged  B. Unchanged.  C. Still flushed, CRT 3s, more cool peripheries. Pulses present.  **D. Less combative – more slurred speech, GCS E2M4V4: Eye opening to pain, Flexion withdrawal to pain, confused**  E. Unchanged | - **EKG/monitors – recognizes wide complex tachycardia. TCA recognized (if not already)** - **Bolus fluid** - Obtains additional IV access - **Sodium bicarbonate 1-2mEq/kg/dose bolus dose – repeat q5 minutes with serial EKG** - **Applies defibrillator pads** - **Considers intubation** - **Calls poison control** - Monitor pulses (possible need for CPR) | - If no bolus fluid by 15 minutes, then Vtach arrest. - If no sodium bicarbonate started by 15 minutes, then Vtach arrest | - TCA toxicity: a-blockade, anticholinergic, and sodium channel blockade. - **Move to phase 3 after completing 2-3 NaHCO3 bolus** - **ECG changes will normalize after 2-3 boluses.** |
| 3. Seizure  Initial vitals:  T 37C \| BP: 90/50 \| HR 160 \| RR 30\| BP: 84/50, SpO2 **83%**  A: Trismus. No stridor  B: GAEB.  C. Flushed, CRT3. Cool peripheries.  D. GCS 3 – patient having GTC  E. Unchanged | - **Benzodiazipine – no response x1** - **Phenobarbitol – will stop seizure** - **Considers intubation** | - No response if phenytoin used for seizure | - RSI – any choice is fine   **Move to phase 4 with initiation of phenobarbital infusion or more than 8 minutes pass (seizure will stop)** |
| 4. Unresponsive and hypotensive  **Seizure will stop – either self limited or because of team intervention**  T: 36 HR: 145, BP: 60/40, Sp02: **75% if on NRB** , RR: 6   1. Unchanged. Snoring sounds 2. Apneic/irregular breathing 3. Cool, pale. Pulses present. CRT 5s 4. GCS 3 0 not responsive. 5. No longer flushed – pale and cool | - **Intubates (if not already done)** - **Consider apneic oxygenation** - **Orders vasopressors (alpha adrenergic – needed to alpha effects)** - **Continue to NaHCO3 bolus**   Call PICU and poison control if not already done | - If not intubated after 5 minutes then PEA arrest - If no pressors started, then PEA arrest. | - **Move to phase 4 with intubation and vasopressors started** - **If doing well then BP fails to improve with norepi – will need intralipid infusion.** |

**Key Debriefing Points:**

1. Value of repeated/serial ECG in TCA toxicity
2. Sodium bicarbonate infusion – how to order and administer – bolus dosing
3. Complications of sodium bicarbonate infusion
4. Differential diagnosis of wideQRS complex tachycardia.
5. Complications of TCA toxicity – seizure, cardiac dysrhythmia and severe hypotension

**Future variations:**

1. TCA overdose and hanging/trauma presentation (double suicide)
2. Polysubstance overdose.
3. Patient arrival in cardiac arrest - PEA, needing CPR.
