# Supplementary material for: Development of an in situ simulation-based continuing professional development curriculum in pediatric emergency medicine
Source: Adv Simul (Lond). 2020 Jul 1;5:12. doi: 10.1186/s41077-020-00129-x (PMC7326623; doi:10.1186/s41077-020-00129-x)
Supplement: Supplementary file 3 — Additional file 3. Sample SimBITS. [file 41077_2020_129_MOESM3_ESM.pdf]

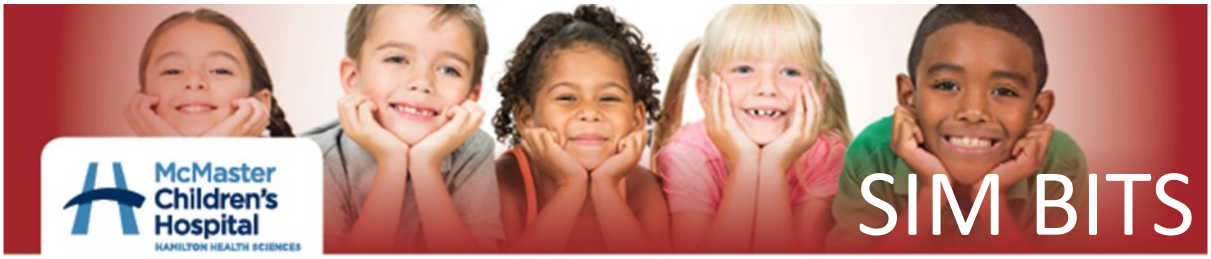

Jan 14th, 2019 **Editors: Mandeep Brar RN, & Leanne Patel Pharm. D**

Focus on Resuscitations in the Peds ED

There are two types of simulations that occur on a monthly basis within the PED; PED specific and trauma. This newsletter will address all the learning opportunities identified during simulation debriefing sessions.

Last summer, we highlighted a hyperkalemia simulation case. Since then , there have been many hyperkalemia SORs identified throughout MCH. A hyperkalemia working group was created. After months of collaboration, a new order set and medication dosing and administration guidelines were created to help standardize the management of acute hyperkalemia.

Order set

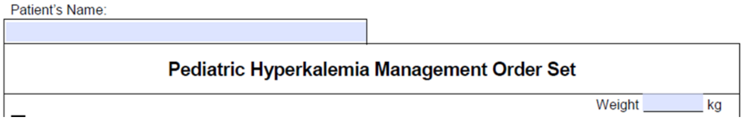
Patient's Name:

Pediatric Hyperkalemia Management Order Set

Weight ______ kg

- Standardizes dosing
- Identify purpose of each medication
- Guides initial management and identify when **PACE activation** in required.
- Provides direction for reassessment post administration

Medication Guide

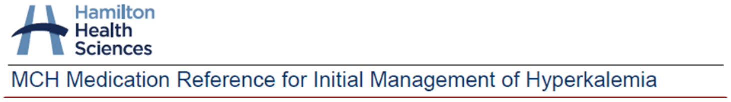
Hamilton Health Sciences

MCH Medication Reference for Initial Management of Hyperkalemia

- Provides preparation and administration instructions

Case Scenario

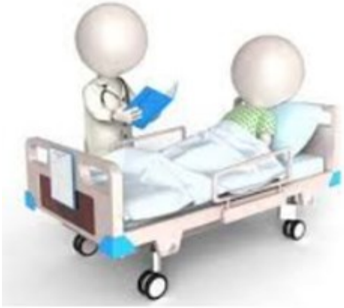

- 7-year-old boy advised by GP to go to ED due to bloodwork results indicating K+ 6.5
- Fever for the last 8 days, fatigue and pale over last 10 days.
- Increasing joint soreness and myalgias. Poor intake in last 7 days with no appetite.
- Sick contacts at home with influenza.
- PMHX: healthy, NKDA, IUTD.

Dx: New Leukemic with Tumor Lysis Syndrome & Severe Hyperkalemia

The focus of this Sim Bits will be on hyperkalemia

Serum potassium is normally maintained between 3.5 -5.0 mmol/L

- Approximately, 98% of potassium is intracellular, 2% of the body's K+ is in the extracellular fluid.
- A gradient exists for the diffusion of K+ from intracellular to extracellular fluid. The gradient is the reverse of that for Na+, which is present in high extracellular concentration & low intracellular concentration.
- Diffusion occurring along both the sodium (Na +) & potassium (K+) gradients is mainly controlled by the sodium- potassium –adenosine triphosphate pump. *Na+/K+ - ATP pump*
- K+ performs an essential role in controlling activity of smooth muscle, skeletal muscle & muscles of the heart. It is also important for normal transmission of electrical signals within the nervous system

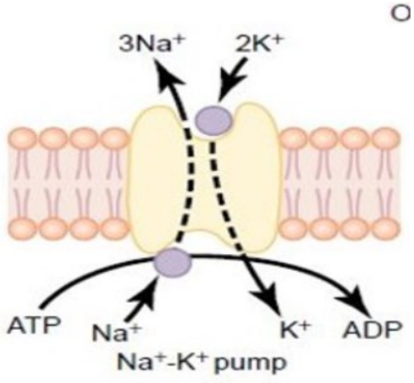

Hyperkalemia is generally defined as a potassium level greater than 6 mmol/L.

[some clinical condition may require treatment with a K+ of 5.5 mmol/L]

Acute Hyperkalemia Management

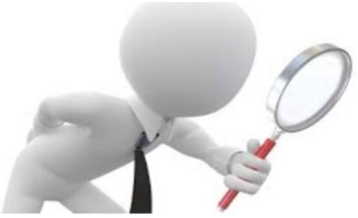

- Ensure sample is not hemolyzed
- Consider repeat K+ STAT
- Obtain ECG
- Consider blood gas, electrolytes, calcium, magnesium, phosphate, CPK, urine K , urine creatinine.
- Stop any source of K+

High levels of K+ can cause progression of EKG changes

- Such as peaked T waves, prolonged PR interval, flattened or absent P waves, wide QRS complex, shortened QT, arrhythmias.

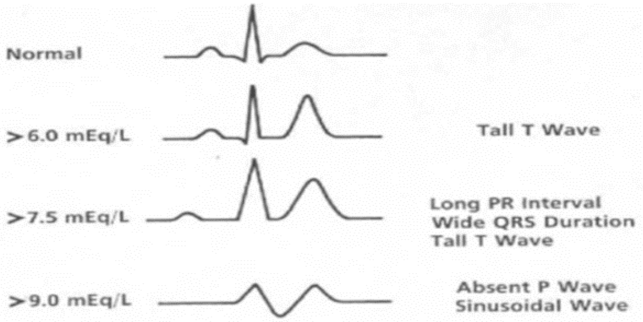

Treatment of hyperkalemia involves:

1. **Stabilizing the cardiac membrane to prevent arrhythmias**
2. **Drive K+ into the intracellular space**
3. **Facilitate removal of K+ from the body.**

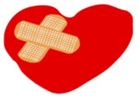

Step 1: Stabilize the cardiac membrane

Calcium

- This type of treatment is temporary to antagonize the effects of hyperkalemia on cardiac muscle & it does not lower the serum K+ level.
- Calcium increases the cellular threshold potential, thereby restoring the normal difference between the resting membrane potential & the firing threshold.
- Indicated if there is widening of QRS, sinusoidal wave, or in hyperkalemic cardiac arrest.
- The 'cardiac membrane stabilizing effects' take minutes.

There are 2 options:

**Calcium GLUCONATE IV**		**Calcium CHLORIDE IV**
preferred option for peripheral administration 50-100 mg/kg (max 2000 mg) IV Dilute to 20 mg/mL 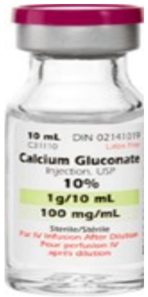	O R	*preferred if liver dysfunction or hemodynamic instability* **Central preferred**: 10-20 mg/kg (max 1000 mg) IV Best diluted to 20mg/mL; can give undiluted 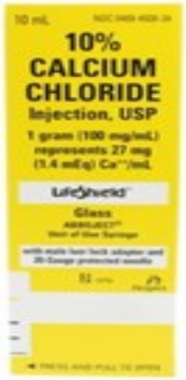
**If unstable: IV push** **If stable: administer on IV pump over 15 minutes**		
Repeat doses q10-20mins if no ECG improvement Will precipitate with sodium bicarbonate – **FLUSH line well** Use with caution in oncology patients with tumor lysis syndrome		

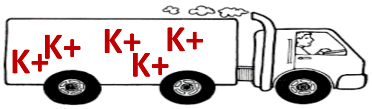

Step 2: Drive K+ into the cell

Salbutamol (Ventolin)

- Beta 2 agonist therapy
- Induce the intracellular movement of K+ via the stimulation of the Na+/K+ ATP pump.
- It can lower K+ level 1mmol/L in about 30 minutes & maintain it for up to 2 hours.

**Due to ease of administration, start salbutamol while other therapies being prepared**		
**Nebulizer (may repeat q 20 min PRN)**<br>**Less than 10kg** dose = 2.5 mg<br>**More than 10kg** dose = 5 mg	OR	**MDI**<br>4 to 10 puffs (may repeat q20min PRN)

Dextrose & Insulin

- Insulin drives K+ into cells and administering glucose prevents hypoglycemia.

DEXTROSE

0.5 g/kg

Give immediately prior to insulin IV over 2-5 minutes

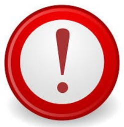

Preparation:

**Less than 10kg**	**D10W**	**5 mL/kg**
**10 kg – 25 kg**	**D50W**	**1mL/kg**<br>**If peripheral**: Dilute equal volume (1:1) with 0.9% NaCl
**Greater than 25 kg**	**D50W**	**1mL/kg**

Administer via CVL if available

A N D

INSULIN

0.1 unit/kg of Regular Insulin (Humulin R), max 10 units

Give IV push immediately

<u>post</u> dextrose

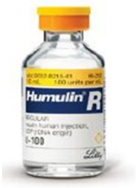

Preparation to make 1 unit/mL solution:

1. Add **1 mL** of Regular insulin (100 units) to 100mL bag of 0.9% NaCl
2. Final concentration of solution = **1 unit/mL**
3. Withdraw required dose from the solution

(i.e. Dose for 15 kg = 1.5 units = 1.5 mL of solution)

Important:

Volume of IV Push Insulin dose will be VERY SMALL

Blood glucose levels should be monitored q1h for at least 6 hours after insulin dose

Consider adding dextrose to maintenance IV fluids.

Sodium Bicarbonate

- Sodium bicarbonate helps drive K+ intracellularly

<table>
<tr><td colspan="2">Sodium Bicarbonate IV 1 mEq/kg (max 50 mEq)</td></tr>
<tr><td colspan="2">If unstable: IV push
If stable: administer on IV pump over 15 minutes</td></tr>
<tr><td>If less than 5kg
Use 4.2% sodium bicarbonate (=0.5 mEq/mL) to avoid cerebral hemorrhage
(if 4.2% not available, dilute 8.4% concentration 1:1 with 0.9% NaCl)
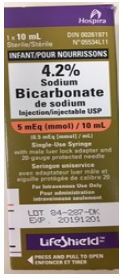
</td><td>If greater than 5 kg
Use 8.4% sodium bicarbonate (=1 mEq/mL)
Give undiluted if CVL; Dilute 1:1 with 0.9% NaCl for peripheral administration
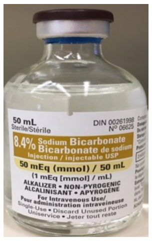
</td></tr>
<tr><td colspan="2">Not compatible with calcium – FLUSH line well</td></tr>
</table>

Step 3: Remove K+ from the body

Furosemide

- K+ wasting diuretic. Helps renal excrete of K+ .

**Furosemide IV**<br>1 mg/kg/dose (max 40 mg)
Give on IV pump over 10 minutes; max rate 0.5mg/kg/min

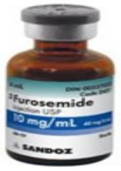

Kayexalate

- Is a large insoluble molecule that binds K+ in the large intestine, where it is excreted in feces.

**Sodium Polystyrene Sulfonate (Kayexalate)**<br>**Note: Not a priority for acute management; peak effects takes 4-6hours**<br>1 g/kg/dose (max PO 15 g/dose; max PR 60 g/dose) q6h prn
Mix 1 gram in minimum 3 mL water (avoid citrus juice). Shake well<br>Do not use if ileus, recent abdominal surgery, perforation or hypernatremia<br>Only to be used in neonates if refractory to other treatment

Dialysis

- If hyperkalemia refractory to therapy. Provides immediate removal of K+.
