# Supplementary material for: Development of an in situ simulation-based continuing professional development curriculum in pediatric emergency medicine
Source: Adv Simul (Lond). 2020 Jul 1;5:12. doi: 10.1186/s41077-020-00129-x (PMC7326623; doi:10.1186/s41077-020-00129-x)
Supplement: Supplementary file 4 — Additional file 4. Interim feedback survey. [file 41077_2020_129_MOESM4_ESM.pdf]

Q1 Have you participated in simulations held in our ED (in situ simulations)? Select all that apply.

Answered: 21 Skipped: 0

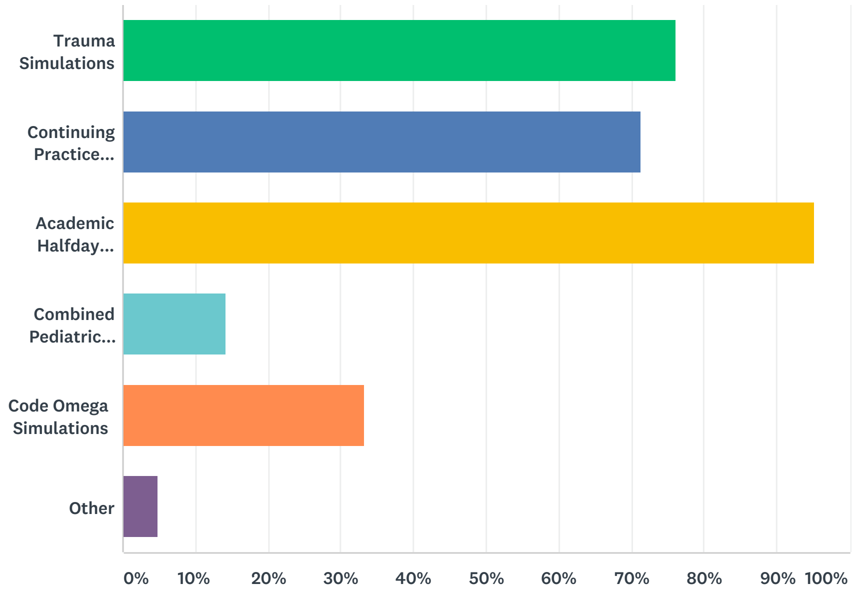

ANSWER CHOICES	RESPONSES	
Trauma Simulations	76.19%	16
Continuing Practice (“Wednesday Staff”) PEM Simulations	71.43%	15
Academic Halfday Supervisor for Resident Simulations	95.24%	20
Combined Pediatric Emergency/NICU/Obs+Gyne Simulations	14.29%	3
Code Omega Simulations	33.33%	7
Other	4.76%	1
Total Respondents: 21		

Q2 The following questions is related to the 18-case Continuing Practice PEM Simulation Curriculum that was distributed. Please review the curriculum and answer the following questionsAs a practicing PEM specialist, what scenarios that you feel will be highest yield for you in maintaining your skills? Select 3 scenarios from the list below.

Answered: 21 Skipped: 0

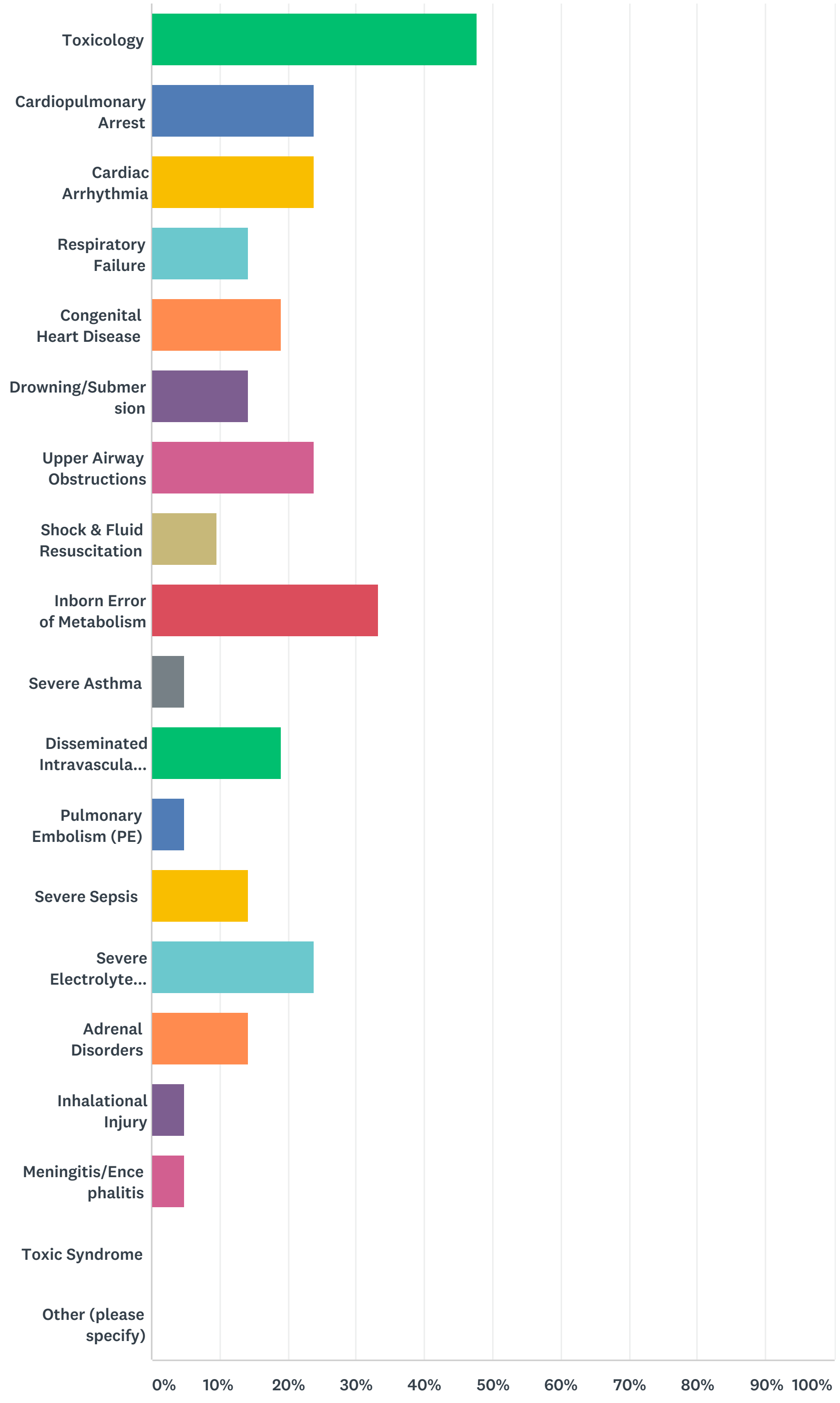

ANSWER CHOICES	RESPONSES	
Toxicology	47.62%	10
Cardiopulmonary Arrest	23.81%	5
Cardiac Arrhythmia	23.81%	5
Respiratory Failure	14.29%	3
Congenital Heart Disease	19.05%	4
Drowning/Submersion	14.29%	3
Upper Airway Obstructions	23.81%	5
Shock & Fluid Resuscitation	9.52%	2
Inborn Error of Metabolism	33.33%	7
Severe Asthma	4.76%	1
Disseminated Intravascular Coagulation	19.05%	4
Pulmonary Embolism (PE)	4.76%	1
Severe Sepsis	14.29%	3
Severe Electrolyte Abnormalities	23.81%	5
Adrenal Disorders	14.29%	3
Inhalational Injury	4.76%	1
Meningitis/Encephalitis	4.76%	1
Toxic Syndrome	0.00%	0
Other (please specify)	0.00%	0
Total Respondents: 21		

Q3 Are there simulation scenarios included in this curriculum that you did not feel needed to be included?

Answered: 21 Skipped: 0

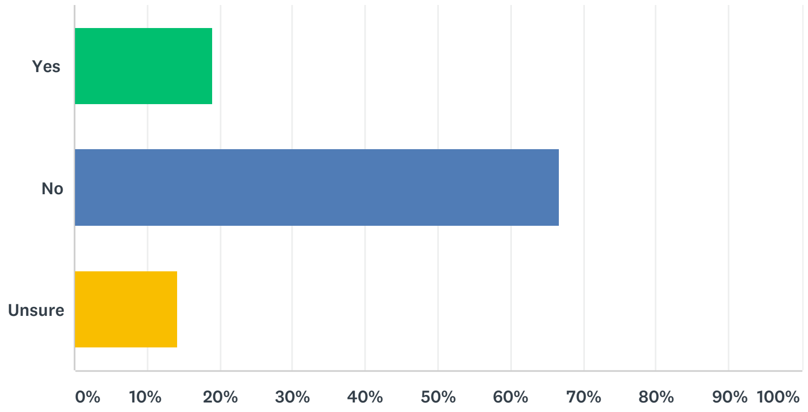

ANSWER CHOICES	RESPONSES	
Yes	19.05%	4
No	66.67%	14
Unsure	14.29%	3
TOTAL		21

Q4 What are the simulation scenarios included in this curriculum that you feel does not need to be included? Select all that apply

Answered: 9 Skipped: 12

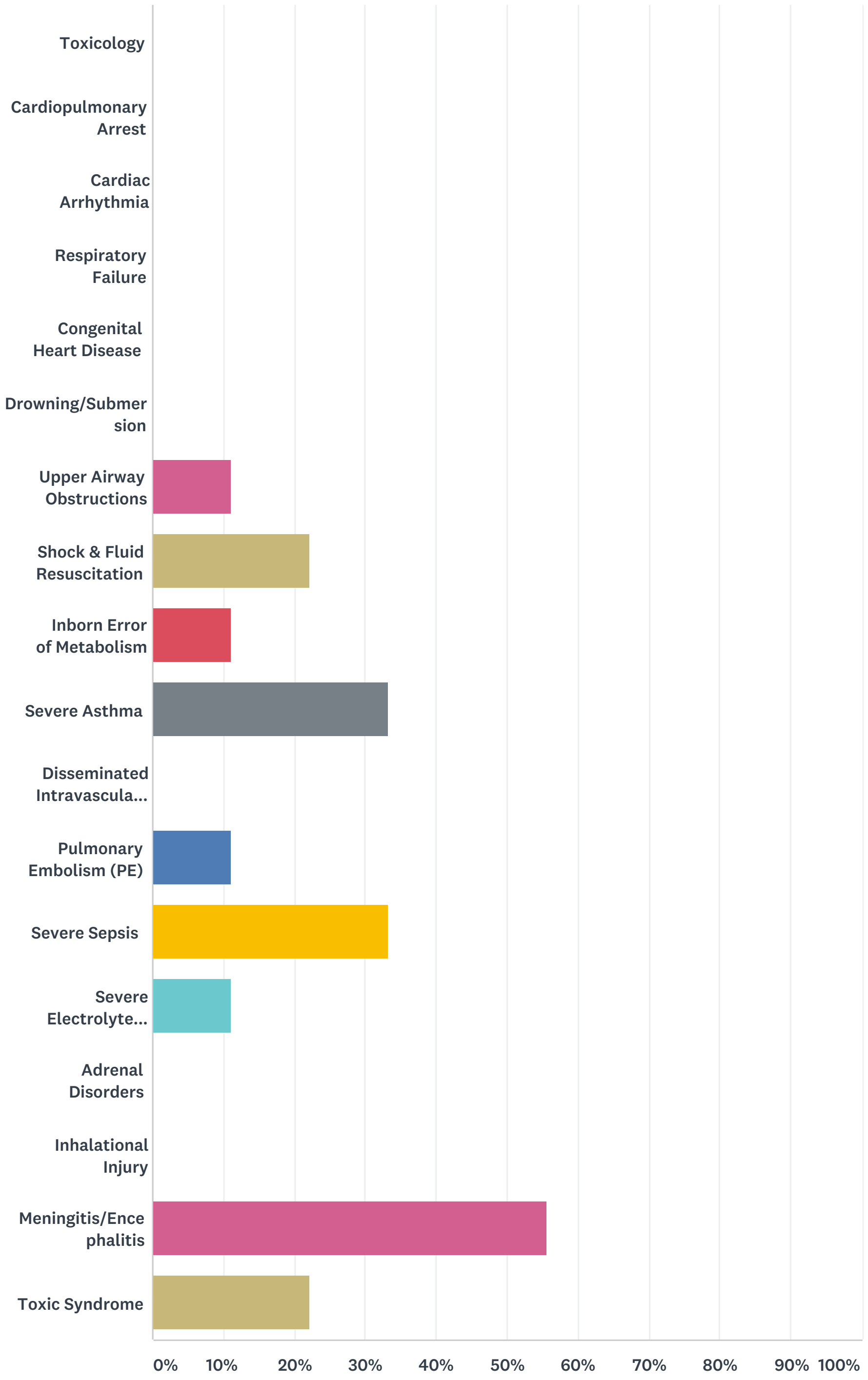

ANSWER CHOICES	RESPONSES	
Toxicology	0.00%	0
Cardiopulmonary Arrest	0.00%	0
Cardiac Arrhythmia	0.00%	0
Respiratory Failure	0.00%	0

Congenital Heart Disease	0.00%	0
Drowning/Submersion	0.00%	0
Upper Airway Obstructions	11.11%	1
Shock & Fluid Resuscitation	22.22%	2
Inborn Error of Metabolism	11.11%	1
Severe Asthma	33.33%	3
Disseminated Intravascular Coagulation	0.00%	0
Pulmonary Embolism (PE)	11.11%	1
Severe Sepsis	33.33%	3
Severe Electrolyte Abnormalities	11.11%	1
Adrenal Disorders	0.00%	0
Inhalational Injury	0.00%	0
Meningitis/Encephalitis	55.56%	5
Toxic Syndrome	22.22%	2
Total Respondents: 9		

Q5 Are there any clinical presentations on the curriculum that were a surprise (i.e. unexpected in its inclusion in our curriculum) to you?

Answered: 21 Skipped: 0

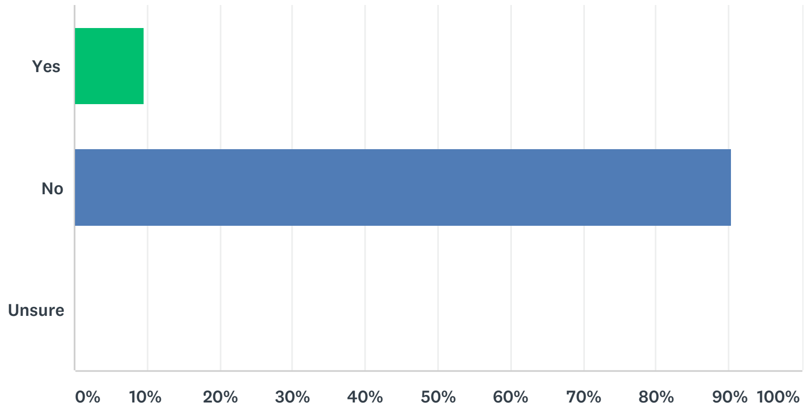

ANSWER CHOICES	RESPONSES	
Yes	9.52%	2
No	90.48%	19
Unsure	0.00%	0
TOTAL		21

Q6 What clinical presentations on the curriculum were unexpected in its inclusion in our curriculum? Select all that apply.

Answered: 11 Skipped: 10

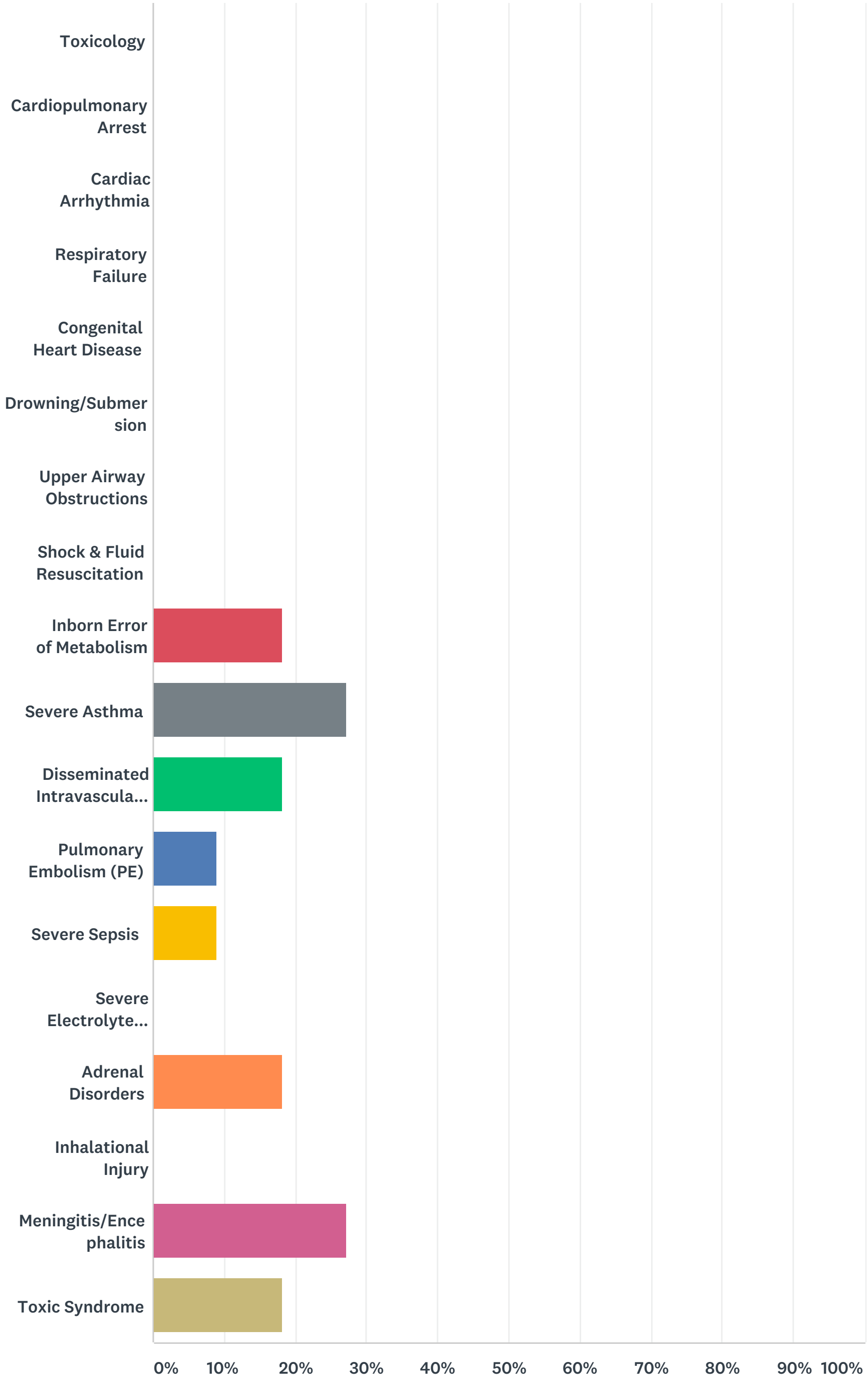

ANSWER CHOICES	RESPONSES	
Toxicology	0.00%	0
Cardiopulmonary Arrest	0.00%	0
Cardiac Arrhythmia	0.00%	0
Respiratory Failure	0.00%	0

Congenital Heart Disease	0.00%	0
Drowning/Submersion	0.00%	0
Upper Airway Obstructions	0.00%	0
Shock & Fluid Resuscitation	0.00%	0
Inborn Error of Metabolism	18.18%	2
Severe Asthma	27.27%	3
Disseminated Intravascular Coagulation	18.18%	2
Pulmonary Embolism (PE)	9.09%	1
Severe Sepsis	9.09%	1
Severe Electrolyte Abnormalities	0.00%	0
Adrenal Disorders	18.18%	2
Inhalational Injury	0.00%	0
Meningitis/Encephalitis	27.27%	3
Toxic Syndrome	18.18%	2
Total Respondents: 11		

Q7 Are there curriculum presentations that you feel should be included in the curriculum, but were not?

Answered: 20 Skipped: 1

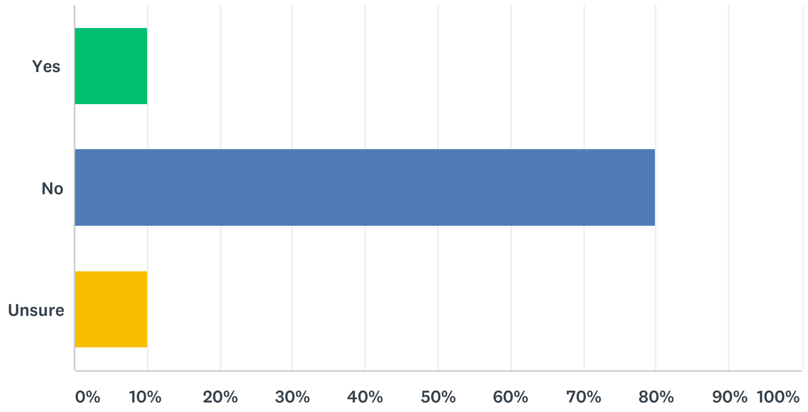

ANSWER CHOICES	RESPONSES	
Yes	10.00%	2
No	80.00%	16
Unsure	10.00%	2
TOTAL		20

Q8 Please list 1-3 scenarios that should be included in future versions of the curriculum that have not been included

Answered: 14 Skipped: 7

Q9 Do you feel that having our shared simulation curriculum (where we share in covering a wide variety of clinical presentations with simulation, with shared knowledge newsletter - SimBITS) will be helpful to you compared to simulation for continuing practice development where simulation cases are created ad hoc (i.e. where cases are made on the fly)?

Answered: 21 Skipped: 0

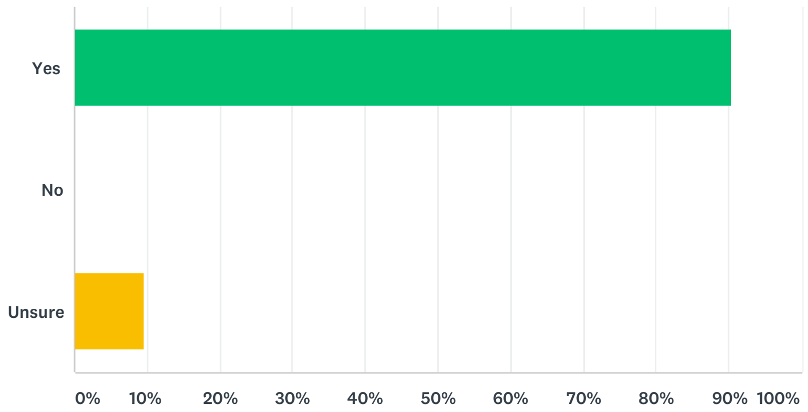

ANSWER CHOICES	RESPONSES	
Yes	90.48%	19
No	0.00%	0
Unsure	9.52%	2
TOTAL		21

Q10 Please the select reasons why a curriculum is valuable compared to ad hoc simulations. A staff simulation curriculum allows for (select all that apply):

Answered: 20 Skipped: 1

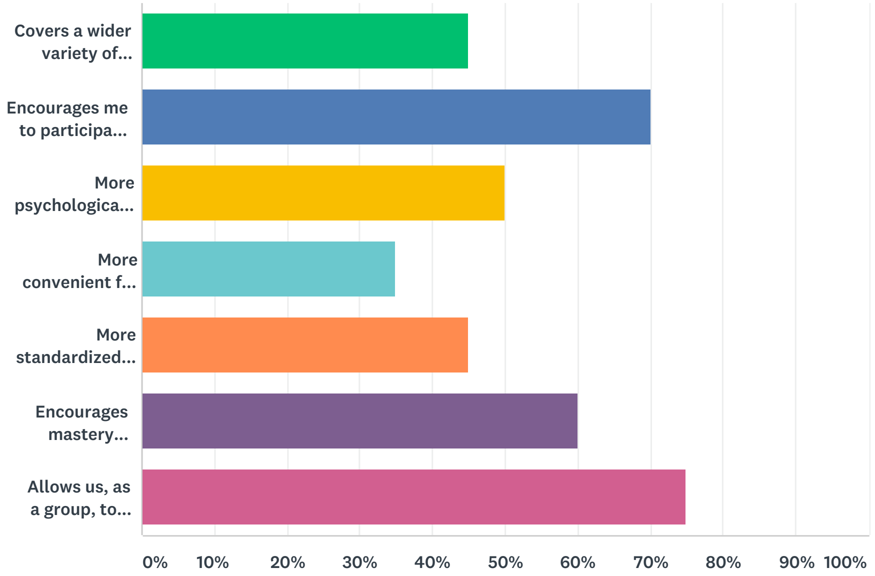

ANSWER CHOICES	RESPONSES	
Covers a wider variety of topics then I would have otherwise considered if I were to self-assess my learning needs	45.00%	9
Encourages me to participate in simulation scenarios that are “outside my comfort zone”	70.00%	14
More psychologically safe and predictable simulation experience	50.00%	10
More convenient for me as a learner to have a prepared list to topics to learn from.	35.00%	7
More standardized, high quality simulation cases to be prepared/curated	45.00%	9
Encourages mastery learning and deliberate practice in PEM.	60.00%	12
Allows us, as a group, to experience a wider variety of clinical presentations and perspectives, developing knowledge through social learning and community of practice.	75.00%	15
Total Respondents: 20		

Q11 Do you envision that having a Simulation Continuing Practice Development (CPD) Curriculum will enhance your learning, compared to PEM CPD Simulations done ad hoc?

Answered: 21 Skipped: 0

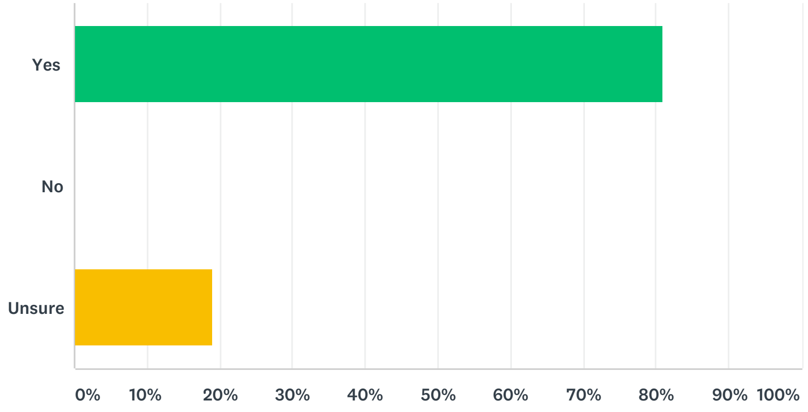

ANSWER CHOICES	RESPONSES	
Yes	80.95%	17
No	0.00%	0
Unsure	19.05%	4
TOTAL		21

Q12 Do you feel you can learn from simulated clinical presentations that you have not directly participated in through SimBITS and other social learning methods?

Answered: 21 Skipped: 0

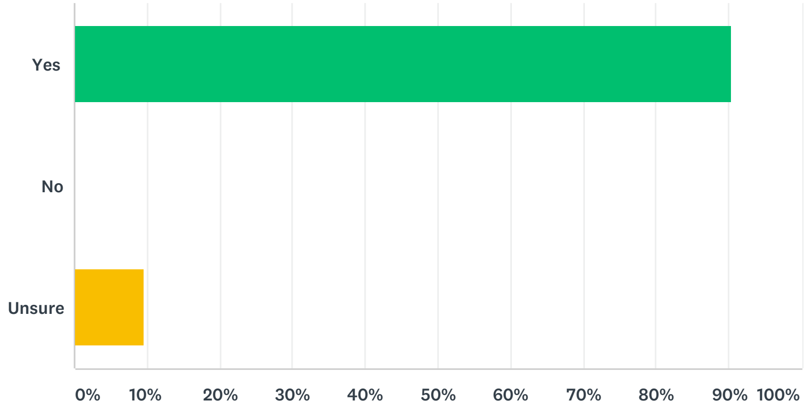

ANSWER CHOICES	RESPONSES	
Yes	90.48%	19
No	0.00%	0
Unsure	9.52%	2
TOTAL		21

Q13 Does having a known Simulation Continuing Practice Curriculum make it more likely that you will participate in simulations for Continuing Practice Development?

Answered: 21 Skipped: 0

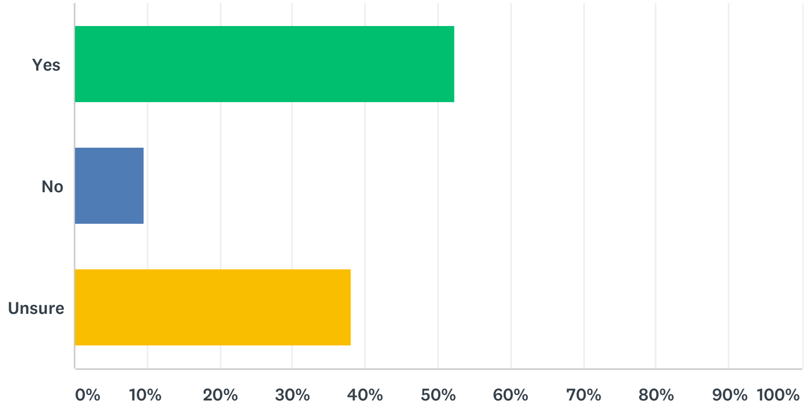

ANSWER CHOICES	RESPONSES	
Yes	52.38%	11
No	9.52%	2
Unsure	38.10%	8
TOTAL		21
